# Fibrillar adhesion dynamics govern the timescales of nuclear mechano-response via the vimentin cytoskeleton

**DOI:** 10.1038/s41563-026-02590-x

**Published:** 2026-04-29

**Authors:** Amy E. M. Beedle, Vivek Sharma, Jorge Oliver-De La Cruz, Anuja Jaganathan, Aina Albajar-Sigalés, F. Max Yavitt, Kaustav Bera, Ion Andreu, Ignasi Granero-Moya, Dobryna Zalvidea, Zanetta Kechagia, Gerhard Wiche, Xavier Trepat, Johanna Ivaska, Kristi S. Anseth, Vivek B. Shenoy, Pere Roca-Cusachs

**Affiliations:** 1 Institute for Bioengineering of Catalonia (IBEC), The Barcelona Institute of Science and Technology (BIST), Barcelona, Spain; 2 Department of Physics, King’s College London, London, UK; 3 Center for Engineering Mechanobiology and Department of Materials Science and Engineering, University of Pennsylvania, Philadelphia, PA USA; 4 Department of Chemical and Biological Engineering, University of Colorado, Boulder, CO USA; 5 BioFrontiers Institute, University of Colorado Boulder, Boulder, CO USA; 6 Instituto Biofisika (UPV/EHU, CSIC), University of the Basque Country, Leioa, Spain; 7 Ikerbasque, Basque Foundation for Science, Bilbao, Spain; 8 Department of Biochemistry, University of Zurich, Zurich, Switzerland; 9 Max Perutz Laboratories, Department of Biochemistry and Cell Biology, University of Vienna, Vienna, Austria; 10 University of Barcelona, Barcelona, Spain; 11 Institució Catalana de Recerca i Estudis Avançats (ICREA), Barcelona, Spain; 12 Centro de Investigación Biomédica en Red en Bioingeniería, Biomateriales y 12 Nanomedicina (CIBER–BBN), Barcelona, Spain; 13 Turku Bioscience Centre, University of Turku and Åbo Akademi University, Turku, Finland; 14 Department of Life Technologies, University of Turku, Turku, Finland; 15 InFLAMES Research Flagship Center, University of Turku, Turku, Finland; 16 Foundation for the Finnish Cancer Institute, Helsinki, Finland

**Keywords:** Molecular biophysics, Cytoskeleton

## Abstract

The cell nucleus is continuously exposed to external signals, of both chemical and mechanical nature. To ensure proper cellular response, cells need to regulate the transmission, timing and duration of these signals. Although such timescale regulation is well described for chemical signals, whether and how it applies to mechanical signals reaching the nucleus is still not fully understood. Here we demonstrate that the formation of fibrillar adhesions locks the nucleus in a mechanically deformed conformation, setting the mechano-response timescale to that of fibrillar adhesion remodelling (~1 h). This process encompasses both mechanical deformation and associated mechanotransduction (such as via YAP), in response to both increased and decreased mechanical stimulation. The underlying mechanism is the anchoring of the vimentin cytoskeleton to fibrillar adhesions and the extracellular matrix through plectin 1f, which maintains nuclear deformation. Our results reveal a mechanism to regulate the timescale of mechanical adaptation, effectively setting a low-pass filter to mechanotransduction.

## Abstract (web summary)

Nuclear deformation and mechano-signalling are regulated by the coupling of vimentin cytoskeleton to the extracellular matrix through fibrillar adhesions, modulating the timescale of mechanotransduction on force removal.

## Main

Mechanical force is a fundamental regulator of cellular behaviour, driving changes in protein conformation and localization, gene expression, and cell function. The inability of a cell to correctly sense force underpins a number of pathologies, including fibrosis and cancer^1,2^. Mechanistically, when a cell receives a mechanical stimulus (such as force application or increased substrate rigidity) from the extracellular matrix (ECM), this triggers a highly coordinated chain of events that propagates the signal across the cytoplasm to the nucleus. This mechanotransduction process includes the growth and maturation of the focal adhesion complexes at the cell surface, and the formation and organization of actin stress fibres, which connect to and mechanically deform the nucleus^3^. In turn, nuclear deformation has a plethora of effects, including, among others, chromatin reorganization^4,5^, signalling at the nuclear envelope^6,7^ and altered nucleocytoplasmic transport dynamics, leading to the nuclear accumulation of mechano-sensitive transcription factors^3,8^.

Overall, mechanotransduction processes involve events that occur at the scale of seconds (such as integrin-mediated reinforcement^9,10^ or altered nucleocytoplasmic transport^3,8^), minutes (such as focal adhesion maturation^11^), hours (such as transcriptional responses or chromatin remodelling^12^) and days or longer (such as tumour growth^13,14^). Some of the longer-lived effects, such as chromatin remodelling, can persist for some time after the mechanical stimulus has ceased, in a phenomenon that has been termed ‘mechanical memory’^15–20^. Even considering such memory effects, mechanical stimulation in physiological conditions may occur at all these timescales, potentially triggering constant, uncontrolled mechano-signalling. For instance, fluctuations in nuclear shape with strains of the order of a few per cent with different implications in mechanotransduction have been reported at the scale of seconds^21,22^, minutes^23^, hours and days^24^. Thus, just as biochemical signalling pathways do^25–28^, it is reasonable to hypothesize that mechanotransduction pathways should have mechanisms controlling the timescale not only of mechanotransduction events but also of the mechanical stimulation itself. However, no such mechanisms have been described, and how they would be enabled by the material properties of cells and nuclei is unclear.

A potential structure that could govern the timescales of mechanical stimulation is the ECM, as it is a fundamental structure transmitting mechanical forces to cells. Further, ECM remodelling has recently been shown to store information of past cellular behaviour. Indeed, fibronectin deposition guides cell migration by generating a physiochemical cue that provides spatial memory^29^, and collagen remodelling promotes the invasion from a mechanically stiff to a soft environment via energy minimization^30^. ECM deposition and remodelling is also a defining feature of cells in a high-rigidity environment^31^. This remodelling occurs concomitantly with the formation of fibrillar adhesions (FBs), which are long-lived α_5_β_1_-integrin-rich adhesions that colocalize with fibronectin fibrils. FBs mature from focal adhesions as they are pulled by actin fibres and get progressively enriched with the protein tensin. From this evidence, it is tempting to hypothesize that ECM remodelling, and FBs, can regulate the timescale of cell mechanical stimulation.

Here we show that actomyosin contractility is required to initiate, but not sustain, nuclear deformation and subsequent mechano-signalling. Instead, nuclear deformation can be sustained simply through the anchoring of the vimentin cytoskeleton to the ECM through FBs. On the loss of mechanical forces, this ECM–vimentin coupling delays mechano-adaptation by maintaining nuclear deformation and the nuclear localization and activity of mechano-sensitive transcription factors. Furthermore, this ECM–vimentin connection buffers high mechanical loads, protecting the nucleus from deformation and damage. Taken together, we unveil a mechanism by which FBs act as a low-pass filter for mechanical stimulation, setting the timescale of response to that of FB remodelling (~1 h).

## Contractility loss reveals nuclear YAP/FB correlation

**Fig. 1:**
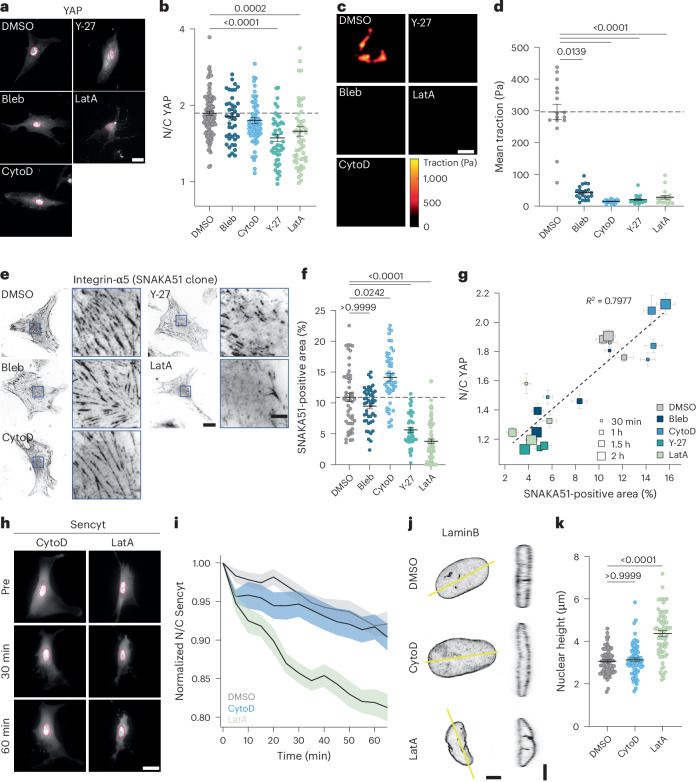
Mechano-sensitive molecules remain nuclear on loss of contractility when FBs are present. **a**,**b**, YAP-stained cells with nuclear outlines (**a**) and N/C ratio quantifications (**b**) after 30 min of indicated pharmacological treatment. Scale bar, 25 μm (*n* = 121/42/62/48/54 cells for DMSO/bleb/cytoD/Y-27/latA from at least three independent experiments; Kruskal–Wallis test, *P* < 0.0001; Dunn’s multiple comparisons test). **c**,**d**, Colour maps of traction forces (**c**) and quantification of mean cell tractions (**d**) after 30 min of pharmacological treatment. Scale bar, 50 μm (*n* = 16/22/21/26/17 cells for DMSO/bleb/cytoD/Y-27/latA from two independent experiments; Kruskal–Wallis test, *P* < 0.0001; Dunn’s multiple comparisons test). **e**,**f**, Integrin α_5_ (SNAKA51 clone)-stained cells (**e**) and quantification of the percentage area under the nucleus occupied by FBs (**f**) after 30 min of the indicated pharmacological treatment. Scale bars, 25 μm; 5 μm (zoomed-in view) (*n* = 52/46/48/45/76 cells for DMSO/bleb/cytoD/Y-27/latA from at least three independent experiments; Kruskal–Wallis test, *P* < 0.0001; Dunn’s multiple comparisons test). **g**, Correlation between N/C YAP ratio and percentage area of FBs under the nucleus for different drug treatments (colour coded) with different incubation times (size coded); at least three independent experiments with a minimum of 41 cells per condition. **h**,**i**, Images of Sencyt (**h**) and quantification of N/C ratio (**i**; normalized to the pretreatment ratio) before and during pharmacological treatment. Scale bar, 25 μm (*n* = 40/33/30 cells for DMSO/cytoD/latA from three independent experiments). **j**,**k**, Confocal images of laminB-stained nuclei (**j**) and quantification of nuclear height after 1 h of the indicated drug treatment (**k**). The yellow line signifies the position of the *z*-plane reslice. Scale bar, 5 µm (*n* = 69/70/70 nuclei for DMSO/cytoD/latA from five independent experiments; Kruskal–Wallis test, *P* < 0.0001; Dunn’s multiple comparisons test). Data are presented as mean ± s.e.m. Source data

**Extended Data Fig. 1:**
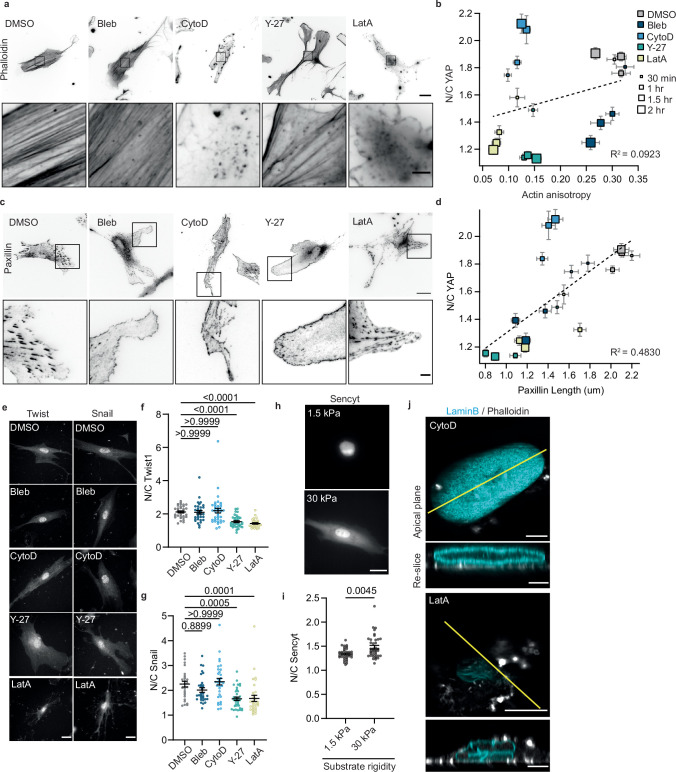
Additional characterization of the effect of pharmacological treatment. **a**. Actin cytoskeleton staining after 2 hour drug treatment; scale bar 25 µm (5 µm in zoomed inset). **b**. Correlation between the N/C YAP ratio and the actin anisotropy for different drug treatments (colour coded) with different incubation time (size coded). 3 independent experiments with a minimum of 41 cells per condition. **c**. Paxillin staining after 2 hour drug treatment; scale bar 25 µm (5 µm in zoomed inset). **d**. Correlation between the N/C YAP ratio and paxillin length for the different drugs at different incubation times. Paxillin data from 3 independent experiments with a minimum of 155 adhesions (31 cells) per condition. **e,f,g**. Twist and Snail stainings (**e**) and quantification of N/C twist (**f**) and N/C snail (**g**) ratio after 30 minute pharmacological treatment. Scale bar 25 µm. (Twist: n = 33/32/38/36/32 cells for DMSO/bleb/cytoD/Y-27/latA and Snail: n = 28/32/32/35/32 cells for DMSO/bleb/cytoD/Y-27/latA. Both from 2 independent experiments; Kruskal-Wallis test with Dunn’s multiple comparison test). **h,i**. Sencyt transfected cells (**h**) and quantification of N/C ratio (**i**) in cells seeded on 1.5 kPa or 30 kPa gels. Scale bar 25 µm. (1.5 kPa, n = 36 cells; 30 kPa, n = 40 cells from 2 independent experiments; Mann-Whitney test). **j**. Confocal images of the nucleus labelled with laminB (cyan) and the actin (grey) after 1 hour pharmacological treatment. The XY image is a single slice at the apical plane on top of the nucleus (scale bar is 10 µm). The re-slice is taken at the yellow line (scale bar is 5 µm). Data are presented as mean +/- SEM. Source data

As the first step to understand how cells adapt to decreased forces, we measured the relocalization of mechano-sensitive transcription factors on the loss of active contractile forces. We first seeded telomerase-immortalized foreskin fibroblasts (TIFF) on fibronectin-coated glass coverslips for 4 h to obtain a highly mechanically active phenotype, with the mechano-sensitive transcription factor YAP localized to the nucleus. We then treated cells for 30 min with different pharmacological inhibitors that interfere with the actomyosin cytoskeleton. We found that there was no change in the nuclear/cytoplasmic (N/C) ratio of YAP on treatment with blebbistatin (bleb; a myosin inhibitor) or cytochalasin D (cytoD; an actin inhibitor), whereas treatment with Y-27632 (Y-27; a ROCK inhibitor) or latrunculin A (latA; an actin inhibitor) triggered a decrease in nuclear localized YAP (Fig. 1a,b). Using traction force microscopy, we verified that all treatments dramatically decreased the active cellular forces (Fig. 1c,d), and therefore, the maintenance of nuclear YAP localization is not explained by sustained cellular force generation. To understand the underlying mechanisms, our first approach was to study known markers of mechanotransduction, such as actin stress fibre organization (assessed through their anisotropy) and focal adhesion length. Surprisingly, the change in localization of YAP on different pharmacological treatments did not correlate with these parameters (Extended Data Fig. 1). Instead, using an epitope-specific integrin-α5 antibody known to be the most specific marker of FBs^32,33^ (SNAKA51), we observed that in conditions under which YAP remained nuclear (bleb and cytoD), the FBs were present, and conditions with the loss of nuclear YAP (Y-27 and LatA) correlated with the loss of FBs (Fig. 1e,f).

To further investigate this relationship, we varied the pharmacological treatment time (30 min to 2 h) and found a good correlation (*R*^2^ = 0.7977) between the presence of FBs and the N/C ratio of YAP, where the loss of fibrils correlates with a loss of nuclear YAP (Fig. 1g). To elucidate whether this phenomenon was specific to the YAP signalling pathway, we performed analogous experiments with other known mechano-sensitive transcription factors twist^34^ and snail^35^. These experiments recapitulated the findings with YAP, indicating that this is a general phenomenon regulating the cytoplasmic relocalization of mechano-sensitive molecules on the loss of active cellular forces (Extended Data Fig. 1).

Of note, a relevant side issue is the reason for the different effects of drugs on FBs. This was due to the different modes of action of the drugs, which depolymerize actin by either binding to actin monomers (LatA)^36^ or to the barbed end of actin filaments (cytoD)^37^. Both treatments abolished the majority of actin stress fibres, as assessed through epifluorescence images and by quantifying the overall actin anisotropy (Extended Data Fig. 1). Both treatments also abolished the actin cap, as assessed by confocal imaging (Supplementary Fig. 1). However, cytoD failed to disassemble basal fibres terminating in FBs (Supplementary Fig. 1). Likely, the barbed end of actin fibres, located within FBs, is shielded by binding partners such as formins^38,39^, impairing binding to cytoD^40^. Similarly, actomyosin associated with FBs may be more shielded from direct binding to bleb^41^, than from the more indirect upstream effect of Y-27 (ref. ^42^). Consistently with this framework, the presence of basal actin and FBs correlated for all drug treatments, and treatment with the formin inhibitor SMIFH2 reduced basal stress fibres, FBs and nuclear YAP levels (Supplementary Fig. 1).

To decouple the contributions of mechano-sensitivity and biochemical signalling regulating the change in molecular localization, we performed experiments using a previously developed mechano-reporter (sensor of nucleocytoplasmic transport—Sencyt). This reporter functions independently of chemical signalling, and instead responds to mechanically induced changes in facilitated and passive nucleocytoplasmic diffusion such that it localizes to the nucleus when it is submitted to force^8,43^ (Extended Data Fig. 1). We transfected cells with Sencyt and treated them with either cytoD or latA, both targeting the actin cytoskeleton but with differential effects on the FBs. On treatment with cytoD, which does not disrupt FBs, the dynamics of Sencyt was indistinguishable from the control dimethyl sulfoxide (DMSO) treatment (Fig. 1h,i). By contrast, treating cells with latA, which disrupts FBs, caused a rapid loss of Sencyt from the nucleus (Fig. 1h,i). The localization of mechano-sensitive molecules such as YAP, snail, twist1 and Sencyt is caused by force transmission to the nucleus and subsequent nuclear deformation. This deformation can be measured as a decrease in nuclear sphericity and an increase in flattening^3,8^. Thus, we sought to understand whether these differences in nuclear localization are associated with changes in nuclear morphology. After 1 h of cytoD treatment, the nuclear height was not significantly different from the control DMSO condition (Fig. 1j,k) despite the total removal of actin stress fibres spanning the nucleus (Extended Data Fig. 1). However, in cells treated for 1 h with latA, the nuclei undergo a significant increase in nuclear height (Fig. 1j,k). Taken together, these results suggest that FBs maintain nuclear morphology and delay the relocalization of mechano-sensitive transcription factors from the nucleus on loss of contractile forces.

## FBs preserve nuclear YAP and shape on contractility loss

**Fig. 2:**
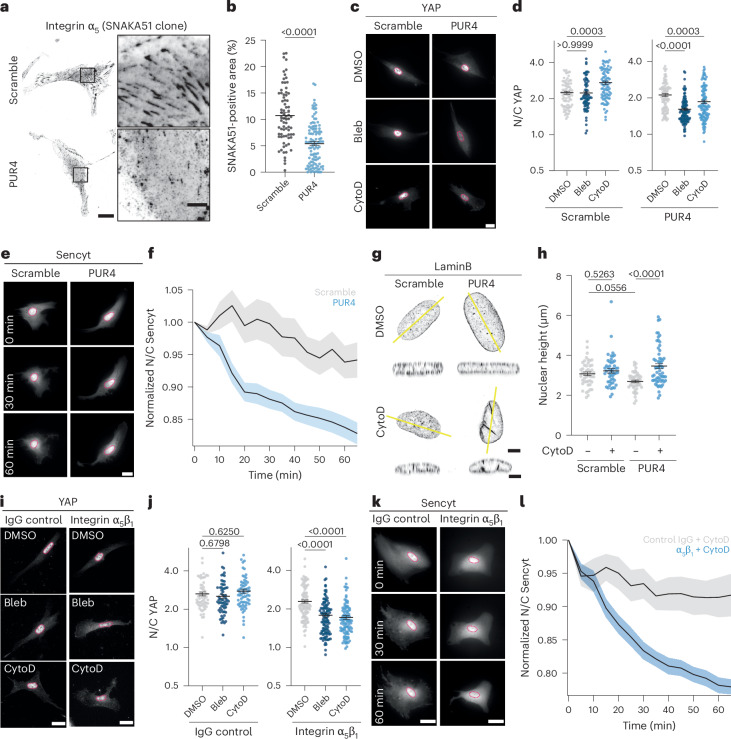
Blocking FBs leads to nuclear exit of mechano-sensitive molecules on loss of contractility. **a**,**b**, Integrin α_5_ (clone SNAKA51) staining (**a**) and quantification of percentage area under the nucleus occupied by FBs (**b**) in cells with scramble and PUR4 peptide. Scale bars, 20 µm; 5 µm (zoomed-in image) (*n* = 74/109 cells for scramble/PUR4 from four independent experiments; two-tailed unpaired *t*-test). **c**,**d**, YAP staining with nuclear outlines (**c**; focused on the nucleus) and quantification of N/C YAP ratio (**d**) in cells treated for 30 min with the indicated pharmacological treatment with scramble and PUR4 peptide. Scale bar, 20 µm (scramble, *n* = 80/74/82 cells for DMSO/bleb/cytoD, and PUR4, *n* = 93/118/110 cells for DMSO/bleb/cytoD, from at least three independent experiments; Kruskal–Wallis test, *P* < 0.0001; Dunn’s multiple comparisons test). **e**,**f**, Sencyt images (**e**) and quantification of N/C ratio (**f**; normalized to the pretreatment ratio) for cells in scramble or PUR4 peptide with cytoD treatment. Scale bar, 20 µm (scramble, *n* = 36 cells, and PUR4, *n* = 44, cells from three independent experiments). **g**,**h**, LaminB-stained nuclei (**g**) and quantification of nuclear height (**h**) for cells in scramble and PUR4 peptide with pharmacological treatment. The yellow line indicates the position of the reslice. Scale bars, 5 µm (scramble, *n* = 46/49 nuclei for DMSO/cytoD, and PUR4, *n* = 48/60 nuclei for DMSO/cytoD, from three independent experiments; two-way analysis of variance (ANOVA) with Tukey’s multiple comparisons test). **i**,**j**, YAP staining (**i**) and quantification of N/C ratio (**j**) in cells blocked with IgG control or α_5_β_1_ integrin antibody and treated with the indicated pharmacological treatment. Scale bars, 20 µm (IgG, *n* = 60/65/67 cells for DMSO/bleb/cytoD, and α_5_β_1_ integrin, *n* = 103/109/103 cells for DMSO/bleb/cytoD, from at least four independent experiments; IgG control; Kruskal–Wallis test, *P* = 0.1318; Dunn’s multiple comparisons test. α_5_β_1_ integrin; Kruskal–Wallis test, *P* < 0.0001; Dunn’s multiple comparisons test). **k**,**l**, Sencyt images (**k**) and quantification of N/C ratio (**l**; normalized to the pretreatment ratio) in cells blocked with IgG control or α_5_β_1_ integrin antibody and treated with cytoD. Scale bars, 20 µm. The solid line represents the average of all trajectories and the shaded area is the standard error (IgG, *n* = 21 cells, and α_5_β_1_ integrin, *n* = 25 cells, from five independent experiments). Data are presented as mean ± s.e.m. Source data

**Extended Data Fig. 2:**
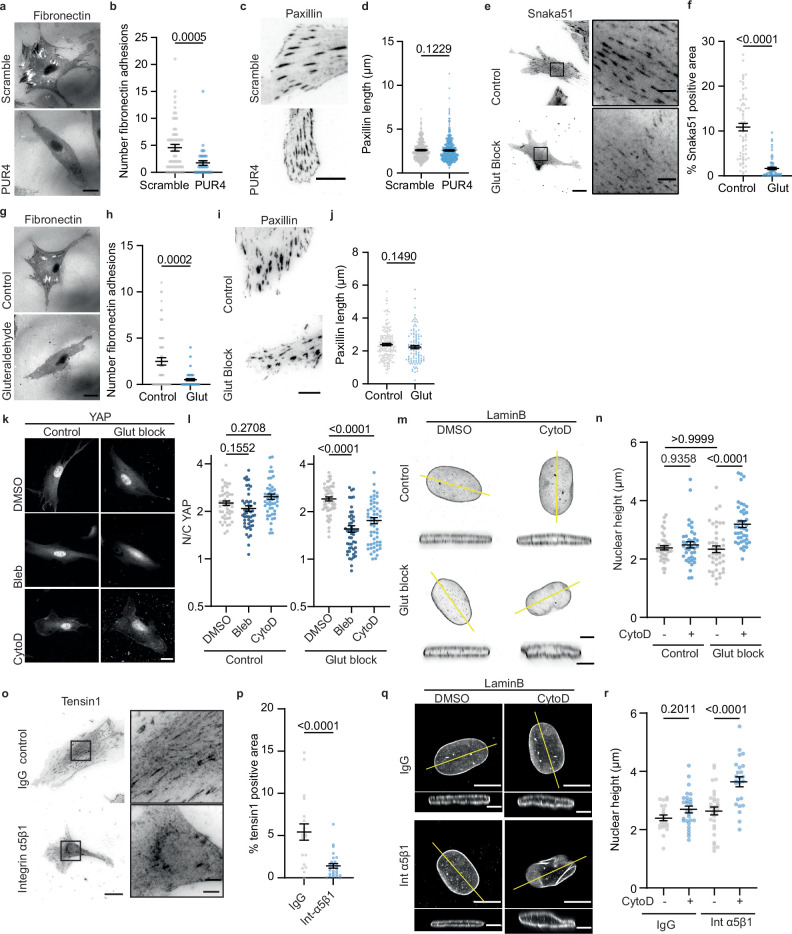
Further characterisation of cell consequences of FB inhibition under contractility loss. **a,b**. Fibronectin stainings (**a**) and quantification of number of fibronectin adhesions (**b**) for cells seeded in scrambled or PUR4 peptide. Scale bar 25 µm. Adhesions were only considered if the intensity was above the background fibronectin intensity. Two-tailed Mann-Whitney test scramble, n = 58 cells and PUR4 n = 44 cells from 2 independent experiments. **c,d**. Paxillin staining (**c**) and analysis of paxillin adhesion length (**d**) for cells seeded with scrambled or PUR4 peptide. Scale bar 10 µm. Two-tailed Mann-Whitney test, scramble n = 440 adhesions, PUR4 n = 524 adhesions from 4 independent experiments. **e,f**. Integrin α5 antibody (clone Snaka51) staining (**e**) and quantification of Percentage area under the nucleus occupied by FBs (**f**) for cells on control and gluteraldehyde blocked glass. Scale bar 25 µm (zoom insert, 5 µm. Two-tailed Mann-Whitney test, control n = 69 cells and glut n = 68 cells from 5 independent experiments). **g,h**. Fibronectin staining (**g**) and analysis of number of fibronectin adhesions (**h**) for cells seeded in control or gluteraldehyde block conditions. Scale bar 25 µm. (Two-tailed Mann-Whitney test, control n = 61 and glut n = 62 cells from 2 independent experiments). **i,j**. Paxillin staining (**i**) and quantification of adhesion length (**j**) in cells seeded on control and gluteraldehyde blocked substrates. Scale bar 10 µm. (Two-tailed Mann-Whitney test, control n = 175 adhesions and glut n = 105 adhesions from 2 independent experiments). **k,l**. YAP staining (**k**) and quantification of N/C ratio (**l**) for cells after 30 min pharmacological treatment with control or gluteraldehyde blocked substrates. Scale bar 20 µm. **(**Control; n = 47/49/49 cells and Glut; n = 52/48/53 cells for DMSO/bleb/cytoD from 3 independent experiments; Kruskal-Wallis test with Dunn’s multiple comparison test). **m,n**. LaminB staining (**m**) and quantification of nuclear height (**n**) for cells on control or glut blocked substrates treated with DMSO or CytoD. Scale bar 5 µm. (control; DMSO n = 40, cytoD n = 39 cells. Glut; DMSO n = 42, cytoD n = 42 cells from minimum of 3 independent experiments; 2-way ANOVA with Tukey’s multiple comparison test). **o,p**. Tensin1 staining (**o**) and analysis of the percentage area of tensin1 under the nucleus (**p**) in cells treated with IgG control or Integrin α5β1 (clone JBS5) blocking antibody (scale bar 25 µm / zoom 10 µm). FBs were marked with tensin1 rather than integrin α5 antibody (clone Snaka51) due to antibody compatibility conflicts as both primary antibodies are raised in mouse. (Two-tailed Mann-Whitney test; control n = 19 cells, integrin α5β1 n = 27 cells, from 2 independent experiments.)**. q,r**. LaminB staining (**q**) and quantification of nuclear height (**r**) in control IgG or integrin α5β1 blocked cells treated with DMSO or cytoD. Central plane of the nucleus, scale bar 10 µm. Reslice of the nuclei corresponding to the yellow line position, scale bar 5 µm. Two-way ANOVA with Tukey correction for multiple comparisons from 3 independent experiments (IgG DMSO, n = 22 cells; IgG cytoD, n = 28; α5β1 DMSO, n = 32; α5β1 cytoD, n = 24). Data are presented as mean +/- SEM. Source data

**Extended Data Fig. 3:**
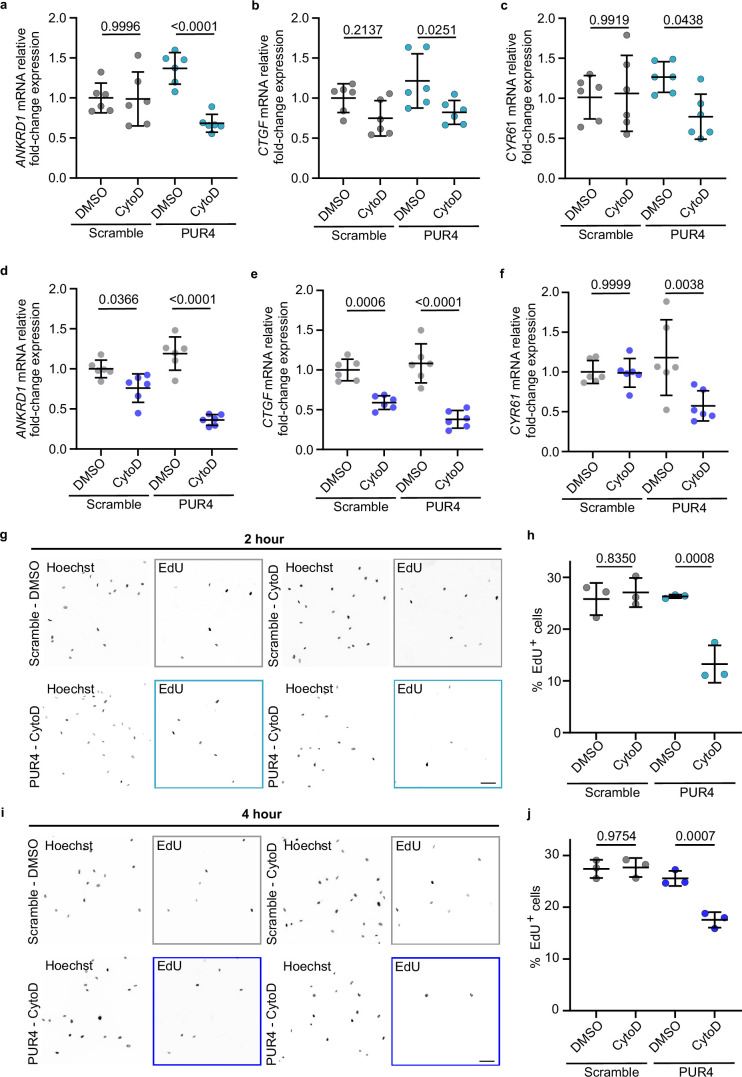
FBs regulate YAP target genes and cell proliferation in response to loss of contractility. **a,b,c**. Relative *ANKRD1* (**a**) *CTGF* (**b**) and *CYR61* (**c**) mRNA expression after 2 hour treatment of DMSO or cytoD. **d,e,f**. Relative *ANKRD1* (**d**) *CTGF* (**e**) and *CYR61* (**f**) mRNA expression after 4 hour treatment of DMSO or cytoD. P-values for all mRNA experiments are calculated with one-way ANOVA from 6 independent experiments. **g,h**. Images of Hoechst and EdU staining (**g**) and quantification of percentage of EdU positive cells (**h**) for cells in the scramble or PUR4 peptide treated for 2 hours with DMSO control or cytoD. Scale bar 100 µm. Each point represents the average value from n = 3 independent experiments (One-way ANOVA statistical test). **i,j**. Images of Hoechst and EdU staining (**i**) and quantification of the percentage of EdU positive cells (**j**) in the scramble or PUR4 peptide treated for 4 hours with DMSO control or cytoD. Scale bar 100 µm. Each point represents the average value from n = 3 independent experiments (One-way ANOVA statistical test). Data are presented as mean +/- SEM. Source data

To test the hypothesis that FBs alter cell response to loss of contractile forces, we impeded the formation of FBs by inhibiting the remodelling of fibronectin. As the first approach, we used the PUR4 (also known as FUD) peptide that binds with high affinity to the N terminus of fibronectin and is a potent inhibitor of the assembly of fibronectin into fibrils^44–46^ (Extended Data Fig. 2). This effectively prevented the formation of FBs (Fig. 2a,b), but did not alter the key aspects of cellular mechanotransduction, including focal adhesion growth, and the nuclear localization of YAP (Extended Data Fig. 2 and Fig. 2c,d). To understand the contribution of FBs on the loss of contractile forces, we exposed cells to either PUR4 or a control scrambled peptide, and treated them with bleb or cytoD. As expected, for the control conditions, these treatments did not trigger a decrease in the N/C ratio of YAP (Fig. 2c,d). However, in the cells lacking FBs, this pharmacological treatment triggered a significant decrease in the N/C YAP ratio (Fig. 2c,d). These differences in YAP were also visible in the transcription of YAP target genes, and in one of the main functional effects of YAP, cell proliferation (Extended Data Fig. 3).

The same trends in YAP localization were obtained when we tracked the mechano-reporter Sencyt over time in cytoD-treated cells, showing the generality of the results beyond YAP (Fig. 2e,f). This change in localization was also associated with a change in nuclear morphology. When the FBs are present (exposure to control peptide), the nuclear height was not affected by cytoD (Fig. 2g,h). By contrast, when FB formation was inhibited (PUR4 exposure), the cytoD treatment increased the nuclear height (Fig. 2g,h).

**Extended Data Fig. 4:**
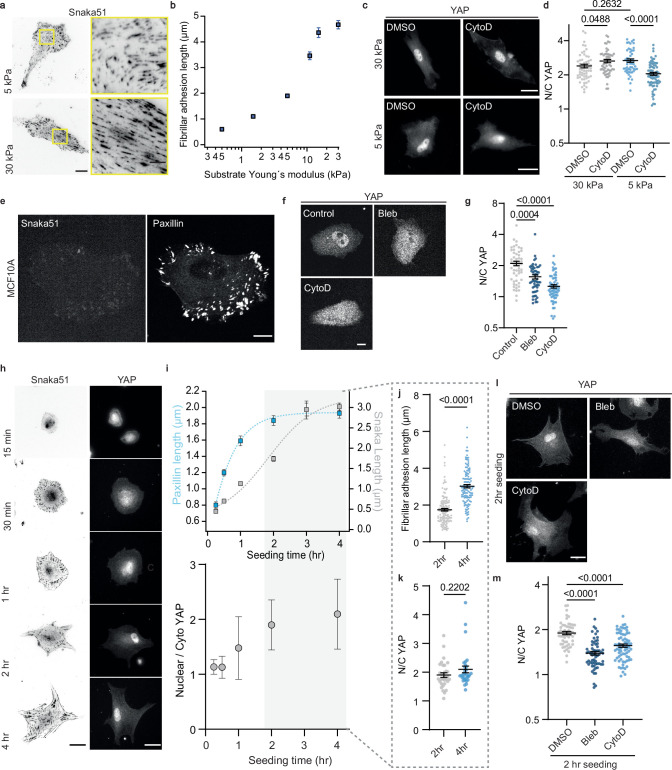
Additional approaches to inhibiting the formation of FBs. **a,b**. Snaka51 staining in cells on 5 kPa and 30 kPa gels (**a**) and quantification of FB length as a function of substrate rigidity (**b**). Scale bar 25 µm / zoom 5 µm. Each data point contains a minimum of 64 FBs from 2 independent experiments. **c,d**. YAP staining (**c**) and quantification of N/C ratio (**d**) in cells seeded on 30 kPa or 5 kPa gels, treated with DMSO or cytoD. Scale bars 25 µm. (30 kPa DMSO, n = 61; 30 kPa cytoD, n = 56; 5 kPa DMSO, n = 52; 5 kPa cytoD, n = 73; two-way ANOVA with Tukey multiple comparison test from 3 independent experiments). **e**. Snaka51 and Paxillin staining of MCF10A cells. Scale bar 15 µm. **f,g**. YAP staining (**f**) and quantification of N/C ratio (**g**) in MCF10A cells after 30 minute of indicated pharmacological treatment. Scale bar 15 µm. (n = 54/53/60 cells for DMSO/bleb/cytoD from 2 independent experiments; Kruskal-Wallis test with Dunn’s multiple comparison test). **h**. Integrin α5 (Snaka51) and YAP staining in cells after indicated seeding time. Scale bar 25 µm.**i**. Upper panel: Analysis of FB length (grey) and focal adhesions length (blue) as a function of seeding time. Data from 2 independent experiments (15 min, n = 89 adhesions; 30 min, n = 115; 1 hr, n = 124; 2 hr, n = 130; 4 hr, n = 120). Lower panel: Analysis of the N/C YAP ratio as a function of seeding time. Data from 2 independent experiments (15 min, n = 30 cells; 30 min, n = 29; 1 hr, n = 33; 2 hr, n = 32; 4 hr n = 31). Circle centre represents mean and error bars are standard deviation. **j**. Quantification of FB length at 2 hr and 4 hr post seeding. (n = 130/120 adhesions for 2 hr/4 hr from 2 independent experiments; Two-tailed Mann-Whitney test). **k**. Analysis of N/C YAP ratio at 2 hr and 4 hr post seeding. (n = 32/31 cells for 2 hr/4 hr from 2 independent experiments; Two-tailed Mann-Whitney test). **l,m**. YAP staining (**l**) and quantification of N/C ratio (**m**) in cells seeded for 2 hours and treated with DMSO, blebbistatin or cytochalasinD. Scale bar is 25 µm. (n = 54/61/73 cells for DMSO/bleb/cytoD from 3 independent experiments; Kruskal-Wallis test with Dunn’s multiple comparison test). Data are presented as mean +/- SEM unless otherwise stated. Source data

To further confirm the role of FBs, we interfered with their formation with six alternative methods. First, we crosslinked fibronectin with glutaraldehyde before cell seeding, which prevents its remodelling and subsequent FB formation^47^. This impaired the formation of fibrillar but not focal adhesions (Extended Data Fig. 2) and led to the same trends in YAP and nuclear height as the PUR4 peptide (Extended Data Fig. 2). Second, we used a blocking antibody against α_5_β_1_ integrins, through which FBs attach to fibronectin^48^. Cells with blocked α_5_β_1_ lacked FBs (Extended Data Fig. 2) but exhibited nuclear localized YAP (Fig. 2i,k). On treatment with bleb or cytoD, cells treated with α_5_β_1_ antibody, but not with a control antibody, rapidly lost the nuclear localization of both YAP (Fig. 2i,j) and Sencyt (Fig. 2k,l). This was accompanied by a change in nuclear morphology (Extended Data Fig. 2). Third, we compared cells seeded on high-rigidity gels (30 kPa), where FBs were formed and YAP was nuclear, to intermediate-rigidity gels (5 kPa), where YAP was already nuclear, but FBs were smaller as described previously^32^ (Extended Data Fig. 4). On treatment with cytoD, the cells on the higher-rigidity gels maintained the N/C YAP ratio, whereas there was a significant reduction in the cells seeded on 5-kPa gels (Extended Data Fig. 4). Fourth, we applied bleb or cytoD to MCF10A mammary epithelial cells, which do not form FBs, and observed a significant reduction in the N/C YAP ratio after 30 min (Extended Data Fig. 4). In all cases, changes in FBs correlated with basal actin (Supplementary Fig. 1).

Additionally, given that FBs form on the maturation of focal adhesions, there should be a time-dependent effect linked to FB formation. To test this, we first probed the timescales of focal adhesion formation, FB formation and YAP localization, and observed that after 2 h of seeding, focal adhesions are formed and YAP is localized to the nucleus, but the FBs were not fully mature (Extended Data Fig. 4). We subsequently inhibited contractility in cells seeded for 2 h and observed a significant change in YAP localization at short timescales (Extended Data Fig. 4). This is in contrast to the lack of effect observed 4 h after cell seeding, when FBs are fully formed (Fig. 1b). Finally, we generated single-cell micropatterns that either prevented or facilitated the maturation of focal adhesions into FBs and only observed the mechano-protective effect in the latter condition (Supplementary Fig. 2). Altogether, these results demonstrate that FBs maintain the localization of mechano-sensitive molecules in the absence of contractility by sustaining a deformed, flat nuclear morphology independently of the actin cytoskeletal network.

## FBs anchor the vimentin network

**Fig. 3:**
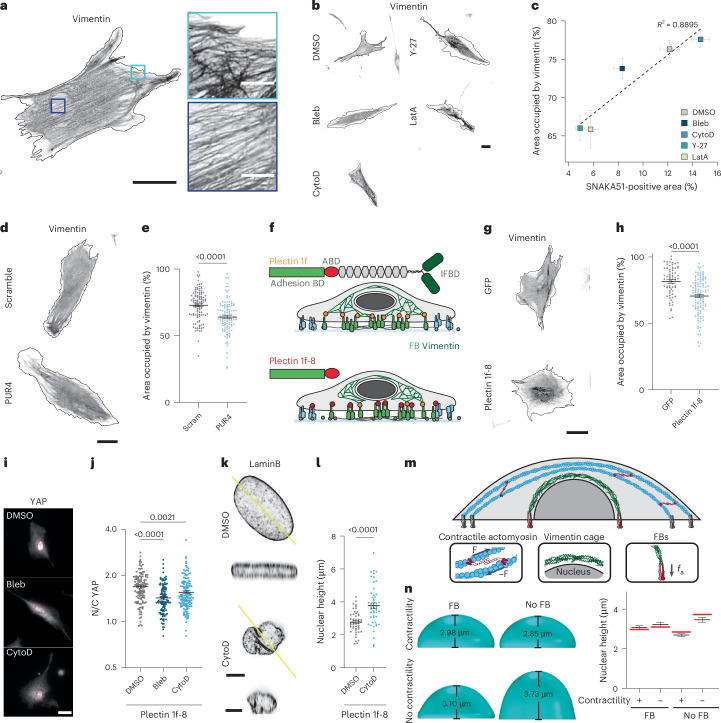
FBs anchor vimentin via plectin 1f and maintain nuclear morphology in absence of contractility. **a**, Vimentin staining (*z* sum) with cell outline (black line). Zoomed-in insets show a single confocal slice at the periphery (light blue) and above the nucleus (dark blue). Scale bars, 25 µm; 5 µm (zoomed-in view). **b**,**c**, Vimentin staining (**b**) and quantification of percentage area of the cell occupied for vimentin versus the percentage area under the nucleus occupied by FBs (**c**) for 1 h of pharmacological treatment. Note: individual fibres are not visible as images are shown as *z* sum of confocal stacks to visualize the overall network spreading. Scale bar, 20 µm (vimentin, *n* = 59/59/59/57/47 cells for DMSO/bleb/cytoD/Y-27/latA from three independent experiments, and FBs, *n* = 123/42/60/48/57 cells for DMSO/bleb/cytoD/Y-27/latA, from at least three independent experiments). **d**,**e**, Vimentin staining with cell outline (**d**; *z* sum) and quantification of the percentage area of the cell occupied by vimentin (**e**) in cells with scramble or PUR4 peptide. Scale bar, 25 µm (*n* = 108/112 cells for scramble/PUR4 from seven independent experiments; two-tailed Mann–Whitney test). **f**, Schematic of the full-length plectin 1f and the truncated plectin 1f-8 proteins. BD, binding domain; IFBD, intermediate filament-binding domain; ABD, actin-binding domain. **g**,**h**, Vimentin staining with cell outline (**g**; *z* sum) and quantification of the percentage area of the cell occupied by the vimentin (**h**) in GPF- and plectin-1f-8-transfected cells. Scale bar, 25 µm (*n* = 67/112 cells for GFP/plectin 1f-8 from at least five independent experiments; two-tailed Mann–Whitney test). **i**,**j**, YAP staining with nuclear outline (**i**; focused on the nucleus) and quantification of N/C ratio (**j**) for plectin-1f-8-GFP-expressing cells treated for 30 min with the indicated pharmacological treatment. Scale bar, 25 µm (*n* = 120/120/132 cells for DMSO/bleb/cytoD from five independent experiments; Kruskal–Wallis test, *P* < 0.0001; Dunn’s multiple comparisons test). **k**,**l**, LaminB-stained nuclei (**k**) and quantification of nuclear height (**l**) in plectin-1f-8-transfected cells treated with DMSO and cytoD. Scale bars, 5 µm (*n* = 48/52 nuclei for DMSO/cytoD from three independent experiments; two-tailed unpaired *t*-test). **m**, Scheme showing the main computational model components. This includes actomyosin contractility driven by myosin motors, and a vimentin network around the nucleus anchored to the substrate via FBs. FBs transmit a vertical resistive force *f*_a_, which prevents nuclear rebounding after the cessation of external forces. **n**, Model predictions for nuclear height with anchored/unanchored vimentin (FBs/no FBs) and in the presence/absence of contractility. Model predictions shown in red and experimental data from Fig. 2h are shown in black for comparison. Data are presented as mean ± s.e.m. Source data

**Extended Data Fig. 5:**
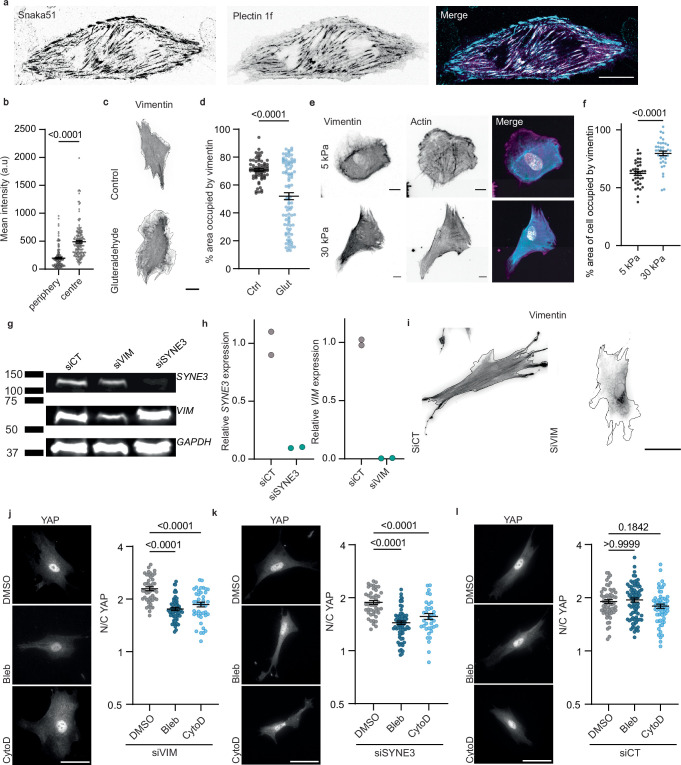
Silencing vimentin or nesprin3 leads to loss of nuclear YAP upon contractility inhibition. **a**. Confocal images (single slice) of cell transfected with plectin 1f-GFP and stained with snaka51 antibody. Scale bar 20 µm. **b**. The mean intensity of plectin 1 f at snaka51 adhesions close to the cell periphery and in the central region of the cell. (n = 160 adhesions from 32 cells from 3 independent experiments; Two-tailed Mann-Whitney test). **c,d**. Confocal mages (z-projection, sum) of vimentin staining (**c**) and quantification of percentage area of the cell occupied by vimentin (**d**) on control or glutaraldehyde blocked substrates; scale bar 25 µm. (n = 68/93 cells for control/glut from 5 independent experiments; Two-tailed Mann-Whitney test). **e,f**. Confocal images (z-projection, sum of 21 slices) of vimentin, actin, and the nucleus (**e**) and quantification of the percentage area of the cell occupied by the vimentin (**f**) in cells seeded on 5 kPa and 30 kPa gels. Scale bars are 10 µm. (n = 36/38 cells for 5 kPa/30 kPa from 3 independent experiments; Two-tailed Mann-Whitney test). **g**. Western blot staining against SYNE3, VIM and GAPDH upon treatment with siRNA targeting CT (control), SYNE3, and VIM. **h**. Quantification of the relative expression of SYNE3 and VIM upon treatment with siRNA targeting CT (control), SYNE3, or VIM. Data from 2 independent experiments. **i**. Vimentin staining 48 hours after treatment with siCT or siVIM. Scale bar 25 µm. **j**. YAP staining in siVIM cells after 30 minute drug treatment. Scale bar 25 µm. Corresponding quantification of N/C YAP ratio (DMSO n = 45, Bleb n = 56, cytoD n = 41 from 3 independent experiments; Kruskal-Wallis test with Dunn’s multiple comparison test.). **k**. YAP staining in siSYNE3 siRNA treated cells after 30 minute treatment with the indicated pharmacological treatment. Scale bar 25 µm. Corresponding quantification of N/C YAP ratio (DMSO n = 45, Bleb n = 69, cytoD n = 36 from at least 2 independent experiments; Kruskal-Wallis test with Dunn’s multiple comparison test.). **l**. YAP staining in siControl siRNA treated cells stained for YAP after 30 minute drug treatment. Scale bar 25 µm. Corresponding quantification of N/C YAP ratio (DMSO n = 61, Bleb n = 73, cytoD n = 61 from 3 independent experiments; Kruskal-Wallis test with Dunn’s multiple comparison test). Data are presented as mean +/- SEM. Source data

To explore the underlying mechanism by which FBs regulate nuclear morphology and mechano-sensitive molecular localization on the loss of contractile forces, we hypothesized that there may be a contribution from cytoskeletal components. Given that YAP remained nuclear even with a severely disrupted actin network (cytoD treatment; Fig. 1), we turned our attention to other cytoskeletal networks. In particular, the vimentin network is known to protect the nucleus in fibroblasts^49^ and has been shown to interact with focal adhesion and FB proteins. Vimentin interacts with FBs through the cytolinker protein plectin isoform 1f (ref. ^50^), suggesting that FBs may regulate the organization of the vimentin intermediate filament (VIF) network. To assess this, we first transfected cells with plectin 1f-GFP and performed stainings against the FB marker SNAKA51 (Extended Data Fig. 5), confirming the presence of plectin 1f in FBs. Then, we assessed the organization of the vimentin network on applying the different perturbations used in Figs. 1 and 2. In control cells, vimentin exhibited a spread morphology, surrounding the nucleus and extending into the cytoplasm (Fig. 3a). In response to pharmacological treatments, we found that conditions with FB loss also exhibited a collapsed vimentin network, measured as a reduction in the percentage area of the cell occupied by vimentin (Fig. 3b,c), with a very high correlation between both parameters (*R*^2^ = 0.8895). On blocking fibronectin remodelling with either the PUR4 peptide (Fig. 3d,e) or glutaraldehyde (Extended Data Fig. 5), the cell area occupied by vimentin decreased. Finally, vimentin spreading was also increased along FBs in response to substrate stiffness (Extended Data Fig. 5). Thus, the ability of vimentin to spread and form a structured cytoskeleton is determined by the cells ability to remodel fibronectin, and the subsequent formation of FBs.

## Vimentin–plectin–FB anchoring sustains nuclear mechanotransduction

Next, we carried out different experiments to assess if the effect of FBs on nuclear mechanotransduction was mediated by vimentin. First, we used siRNA to deplete either vimentin or nesprin 3, which links vimentin to the nuclear lamina^51^. In these conditions, YAP remained nuclear in the absence of drug treatments (Extended Data Fig. 5). However, actomyosin disruption with either bleb or cytoD decreased YAP nuclear localization in both vimentin- and nesprin-3-depleted cells, but not in cells treated with a control non-targeting siRNA (Extended Data Fig. 5). This shows that the vimentin network and its connection to the nucleus is not required to initiate nuclear mechanotransduction, but it is necessary to sustain it in the absence of actomyosin contractility.

**Extended Data Fig. 6:**
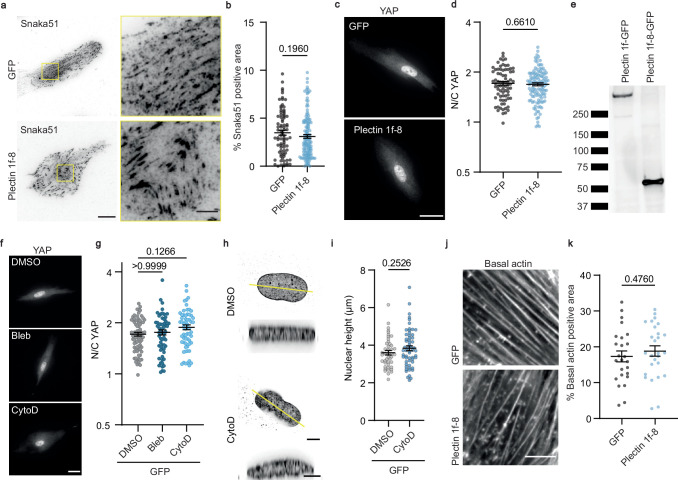
Additional characterisation of the effect of plectin 1f-8 expression. **a,b**. Snaka51 staining (**a**) and quantification of percentage area under the nucleus occupied by FBs (**b**) for cells transfected with GFP or plectin 1f-8-GFP; scale bar 25 µm/ zoom 5 µm. (n = 83/134 cells from 2 independent experiments; Two-tailed Mann-Whitney test). **c,d**. YAP staining (**c**) and quantification of N/C ratio (**d**) for cells expressing GFP or plectin 1f-8-GFP. Scale bar 25 µm. (GFP n = 76, plectin 1f-8-GFP n = 120, from at least 3 independent experiments; Two-tailed unpaired t-test). **e**. Western blot confirming expression of plectin 1f-GFP and plectin 1f-8-GFP, using anti-GFP antibody. Bands correspond to the expected molecular weights: Plectin 1f-GFP at ~ 533 kDa and plectin 1f-8-GFP at ~ 59.3 kDa. Western blot is representative of two independent experiments. **f,g**. YAP staining (**f**) and quantification of N/C ratio (**g**) in control GFP transfected cells subjected to 30 minute pharmacological treatment stained with YAP antibody- Scale bar 25 µm. (n = 76/61/62 cells for DMSO/bleb/cytoD from 3 independent experiments; Kruskal-Wallis test with Dunn’s multiple comparison test). **h,i**. LaminB staining (**h**) and quantification of nuclear height (**i**) in GFP transfected cells treated with DMSO or CytoD. Scale bar 5 µm. (n = 44/58 cells for DMSO/cytoD from 3 independent experiments; Two-tailed Mann-Whitney test). **j,k** Actin images (single confocal slice) of the basal plane of cells (**j**) and quantification of the percentage area under the nucleus occupied by basal actin fibres (**k**) in cells transfected with GFP or plectin 1f-8; Scale bar 5 µm. (GFP n = 25 cells, plectin 1f-8 n = 27 cells from 3 independent experiments. Two-tailed unpaired t-test). Data are presented as mean +/- SEM. Source data

Second, we assessed if the effects of vimentin were mediated specifically by its connection to FBs. To investigate this, we transfected cells with a truncated version of plectin 1f, which contains the FB-binding *N*-terminal domain but lacks the intermediate filament-binding C-terminal domain^50^ (plectin 1f-8) (Fig. 3f). Cells overexpressing plectin 1f-8-GFP formed FBs normally (Extended Data Fig. 6), but exhibited reduced vimentin spreading compared with cells transfected with GFP alone (Fig. 3g,h). Thus, plectin 1f-8 functions as a dominant negative, probably by displacing some of the endogenous plectin 1f and reducing the connectivity between the FBs and vimentin. Consistently with vimentin and nesprin 3 depletion, plectin 1f-8 overexpression did not affect the N/C YAP ratios in control untreated cells (Extended Data Fig. 6), but it abolished their ability to retain nuclear YAP on contractility inhibition (Fig. 3i,j and Extended Data Fig. 6). Furthermore, on treatment with cytoD, which destroys the actin cap, cells expressing GFP alone maintained their nuclear morphology (Extended Data Fig. 6), but in plectin-1f-8-expressing cells, the nuclear height significantly increased (Fig. 3k,l). Of note, plectin-1f-8-expressing cells exhibited unaltered levels of basal actin connected to FBs, further confirming that the effects on nuclear mechanotransduction are explained by interactions with vimentin and not with actin (Extended Data Fig. 6). Thus, a vimentin network properly anchored by plectin 1f is able to sustain a deformed nuclear morphology in the absence of contractile forces and a compressive actin cap, maintaining the localization of the mechano-sensitive transcription factor YAP.

To test whether vimentin anchorage alone can preserve nuclear shape, we asked if a perinuclear vimentin cage, when linked to the matrix via FBs, is sufficient to sustain nuclear flattening in the absence of contractile forces. Both stresses applied by actomyosin contraction^52,53^ and cell and nuclear stiffnesses^54,55^ are in the approximate order of 10^2^–10^3^ Pa, and thus, actomyosin contraction can apply significant strains on the nucleus leading to micrometre-scale deformations, as seen, for instance, during cell migration^56^. These strains could be modulated by vimentin–ECM connections via FBs. To assess this more rigorously, we generated a mechanical model considering the key elements involved. The model (Methods) considers a contractile actomyosin network anchored to the cell periphery via focal adhesions. A vimentin network spans the nucleus and is anchored to FBs via adhesive interactions, which transmit force to the substrate (Fig. 3m). In conditions lacking FBs, this adhesive force is set to zero, effectively decoupling vimentin from the extracellular environment. We first allowed the contractile actin network to form, leading to high compressive strains and a flattening of the nucleus (Fig. 3n). Regardless of whether vimentin is anchored, we observed a similar nuclear morphology that is consistent with the experimental observations. We subsequently removed contractility from the model and observed the effect on nuclear morphology, which also serves as a proxy for changes in YAP. In the condition where vimentin is anchored to FBs, the adhesive force prevents any change in nuclear morphology. This is because once the FBs are engaged with the vimentin cage, the interactions persist even when contractility is abrogated. Although contractility is not needed to sustain these bonds, it is essential to initiate their formation. By contrast, in the case where vimentin is not anchored, we observe a significant rounding of the nucleus (Fig. 3n). This is consistent with the experimental results and demonstrates that vimentin anchoring to the substrate is sufficient to maintain a compressed nuclear morphology in the absence of active contractile forces.

## ECM–vimentin coupling tunes mechano-adaptation timescales

**Extended Data Fig. 7:**
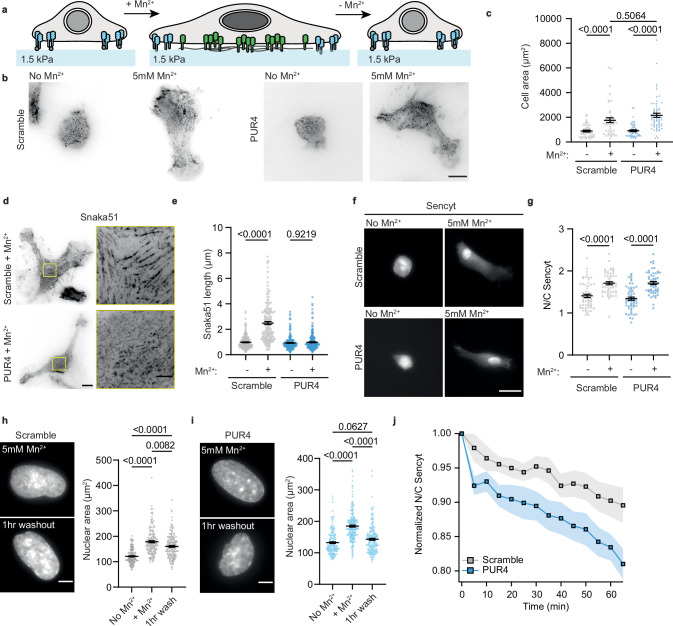
Cellular adaptation timescales depending on fibrillar adhesion formation. **a**. Schematic of experimental design. Cells on 1.5 kPa gels are treated with Mn^2+^ to activate integrin α5β1, inducing cell spreading and a mechanically active phenotype. Mn^2+^ is then washed out, and cell adaptation is monitored. **b**. Snaka51 staining in cells seeded on 1.5 kPa gels in the absence or presence of 5 mM Mn^2+^, in scramble or PUR4. Scale bar 20 µm. **c**. Quantification of cell area for scramble and PUR4 in the absence or presence of Mn^2+^. (Scramble n = 43, scramble with Mn^2+^ n = 45, PUR4 n = 57, PUR4 with Mn^2+^ n = 64 cells, from 3 independent experiments; 2-way ANOVA with Tukey’s multiple comparison test). **d,e**. Snaka51 staining (**d**) and quantification of FB length (**e**) in cells treated with Mn^2+^ in scramble or PUR4 peptide. Scale bar 20 µm / 5 µm zoom. (Scramble n = 240 adhesions (48 cells), Scramble with Mn^2+^ n = 170 (34 cells), PUR4 n = 230 (46 cells), PUR4 with Mn^2+^ n = 280 (56 cells); from 3 independent experiments; 2-way ANOVA with Tukey’s multiple comparison test). **f,g**. Sencyt transfected cells (**f**) and quantification of N/C ratio (**g**) in cells seeded on 1.5 kPa gels in absence or presence of Mn^2+^ for scramble and PUR4 peptide. Scale bar 20 µm. (Scramble n = 61, scramble with Mn^2+^ n = 55, PUR4 n = 57, PUR4 with Mn^2+^ n = 55 cells from 3 independent experiments; 2-way ANOVA with Tukey’s multiple comparison test). **h,i**. Nuclear area of cells on 1.5 kPa gels in the absence, presence, or after 1 hour washout of Mn^2+^ in the scramble peptide (**h**) and PUR4 peptide (**i**). (Scramble: n = 157/151/160 cells for No-Mn^2+^/With- Mn^2+^/ Mn^2+^-washout from 5 independent experiments; PUR4: n = 164/203/192 cells for No-Mn^2+^/With- Mn^2+^/ Mn^2+^-washout from 5 independent experiments; Kruskal-Wallis test with Dunn’s multiple comparisons test). **j**. The change in N/C ratio of Sencyt (normalized to pre-washout value) upon removal of the Mn^2+^ for scramble (grey) and PUR4 (blue) condition (Scramble n = 37 cells, PUR4 n = 35 cells from 3 independent experiments). Data are presented as mean +/- SEM. Source data

Thus far, we have shown that the connectivity between the FBs and vimentin network delays the loss of mechanical signals on inhibition of cellular contractility. Next, we asked whether this mechanism would also determine the timescales by which cells adapt from a high-rigidity to low-rigidity mechanical environment. Indeed, by maintaining a mechanically active phenotype, FBs could delay the timescales of adaptation from a stiff-to-soft conversion. As an initial experiment, we treated cells seeded on low-rigidity polyacrylamide gels (1.5 kPa) with Mn^2+^, which activated α_5_β_1_ integrins and initiated cell spreading, FB formation and nuclear localization of Sencyt, mimicking the mechanical activation of stiff gels (Extended Data Fig. 7). On removal of Mn^2+^, PUR4-treated cells (with blocked FBs) decreased the nuclear area and nuclear localization of Sencyt, faster than cells treated with the scrambled peptide (Extended Data Fig. 7).

**Fig. 4:**
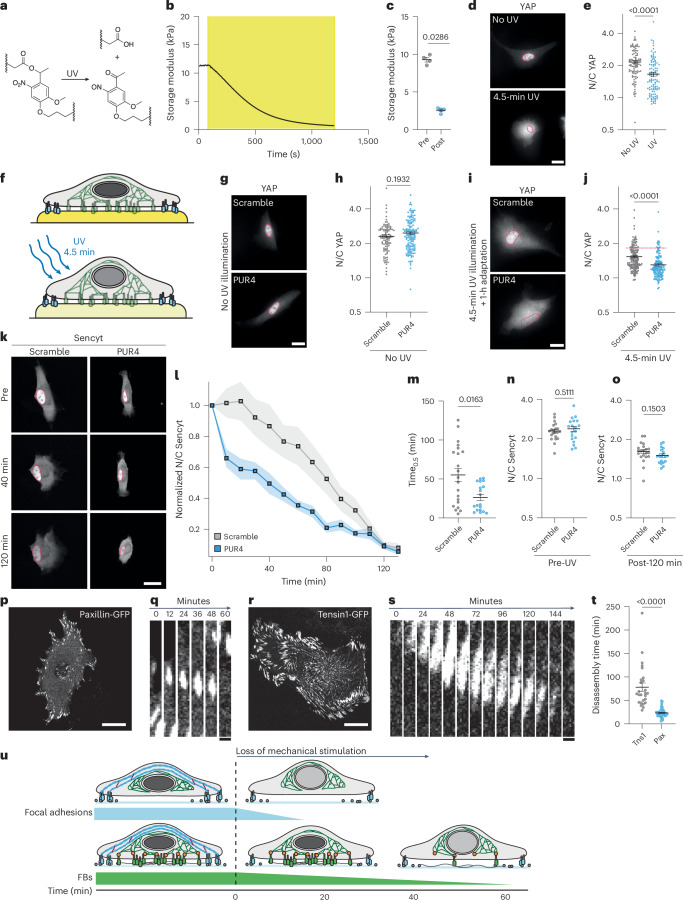
ECM–vimentin coupling delays mechano-adaptation to a soft environment. **a**, Chemical structure of the photodegradable compound. **b**, Storage modulus of the photodegradable gel, which softens on illumination with UV light (yellow region; 365 nm, 10 mW cm^−2^). Trace representative of three gels. **c**, Quantification of the gel storage modulus before and after 5 min of UV exposure (*n* = 4 gels; two-tailed Mann–Whitney test). **d**,**e**, YAP staining with nuclear outlines (**d**) and N/C ratio quantifications (**e**) in cells seeded on gels not softened, or softened with 4.5 min of UV illumination before cell seeding. Scale bar, 20 µm (*n* = 98/107 cells for no UV/UV from three independent experiments; two-tailed Mann–Whitney test). **f**, Schematic of the experimental setup, where cells are seeded on photodegradable gels for 4 h before UV-induced softening. **g**,**h**, Examples (**g**) and N/C ratio quantifications (**h**) of YAP on photodegradable gels without UV exposure in scramble and PUR4 conditions. Scale bar, 20 µm (*n* = 100/141 for scramble/PUR4 from three independent experiments; two-tailed Mann–Whitney test). **i**,**j**, Examples (**i**) and quantification (**j**) of YAP on photodegradable gels 1 h after 4.5 min of UV exposure for scramble and PUR4 conditions. Scale bar, 20 µm. The red line is the mean N/C YAP ratio for cells on non-degradable gels with the same UV conditions (*n* = 170/144 cells for scramble/PUR4 from three independent experiments; two-tailed Mann–Whitney test). **k**,**l**, Examples of confocal slice (**k**) and N/C ratio quantifications (**l**; normalized to the initial and final ratio) of Sencyt on photodegradable gels before and after UV illumination for the scramble and PUR4 conditions. Scale bar, 20 µm (*n* = 21/19 cells for scramble/PUR from three independent experiments). **m**, Time taken for the Sencyt ratio to fall below 50% of the initial value for scramble and PUR4. *n* = 21/19 cells for scramble/PUR from three independent experiments; two-tailed Mann–Whitney test). **n**,**o**, N/C Sencyt ratio for cells seeded on photodegradable gels before (**n**) and after (**o**) UV illumination (*n* = 21/19 cells for scramble/PUR from three independent experiments; unpaired two-tail *t*-test). **p**–**s**, Single-slice confocal images of cell expressing paxillin-GFP (**p**) or tensin1-GFP (**r**) seeded on glass, along with examples of adhesions at 12-min intervals (**q** and **s**). Scale bars, 20 µm (main images); 1 µm (adhesion examples). **t**, Adhesion disassembly time of paxillin and tensin1 adhesions (paxillin, *n* = 47 adhesions, 11 cells, five independent experiments; tensin1, *n* = 26 adhesions, 10 cells, four independent experiments; two-tailed Mann–Whitney test). **u**, Schematic showing how adhesions dynamics and anchoring of vimentin determines the timescales of nuclear response on a loss of mechanical stimulation. Data are presented as mean ± s.e.m. Source data

**Extended Data Fig. 8:**
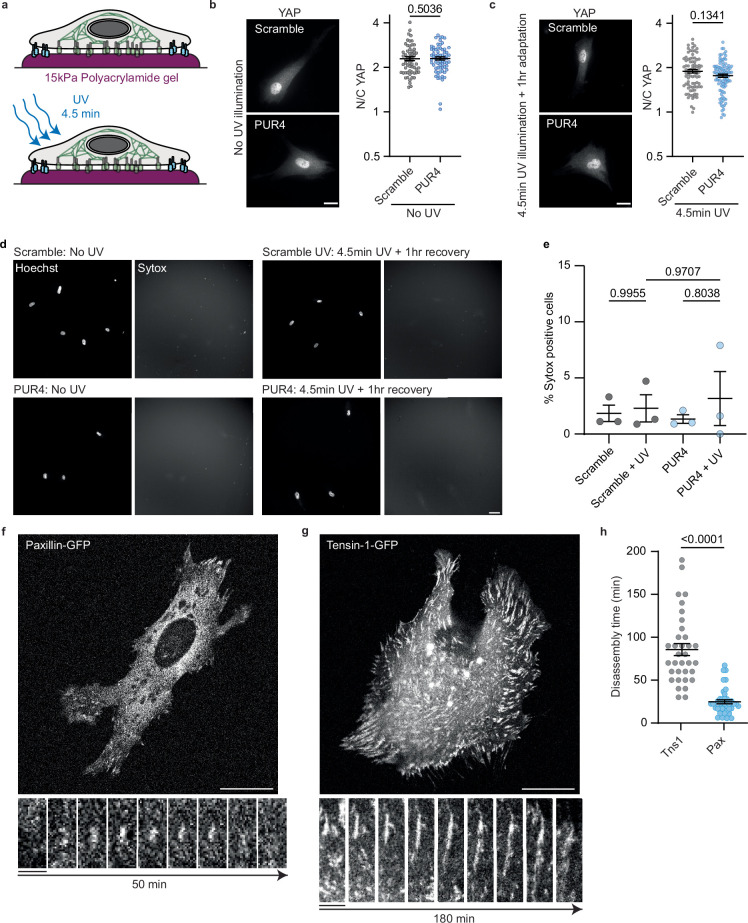
Additional characterisation of cells on elastic gels. **a**. Schematic of experimental set-up, where cells are seeded on 15 kPa polyacrylamide (PAA) gels prior to UV illumination in the presence of the scramble or PUR4 peptide. **b**. YAP staining and quantification of N/C ratio in cells seeded on 15 kPa PAA gels without UV exposure, cultured in scramble or PUR4 peptide. Scale bar 20 µm. Two-tailed Mann-Whitney test from 2 independent experiments, Scramble n = 60 cells, PUR4 n = 68 cells. **c**. YAP staining and quantification of N/C ratio in cells seeded on 15 kPa PAA gels with 4.5 min *in-situ* UV exposure followed by 1 hour adaptation time, cultured in scramble or PUR4 peptide. Scale bar 20 µm. Two-tailed Mann-Whitney test from 2 independent experiments, Scramble n = 83 cells, PUR4 n = 98 cells. **d,e**. Sytox (a marker of cell death) staining (**d**) and quantification of the percentage of Sytox positive cells (**e**) before and after UV treatment (scale bar 50 μm). Each point is the percentage from an experimental replicate, from n = 3 independent experiments (Two-way ANOVA with Šídák’s multiple comparisons test). **f**. Cell expressing Paxillin-GFP on a 15 kPa PAA gel. Lower panel is a time series of a single adhesion, total series is 50 mins. (Scale bar 20 μm, zoom 2 μm). **g**. Tensin-1-GFP expressing cell on PAA gel. Lower panel is a timeseries of a single adhesion, total series is 180 min. Scale bar 20 μm, zoom 2 μm). **h**. Analysis of adhesion disassembly timescales on 15 kPa gels (Tns1 n = 34 adhesions from 8 cells, pax n = 42 adhesions from 9 cells. Data from 2 independent experiments; Two-tailed Mann-Whitney test). Data are presented as mean +/- SEM. Source data

These experiments suggest a delay in mechano-adaptation, but do not fully mimic a change in substrate rigidity. To improve this, we fabricated stiff hydrogels that contain a photocleavable crosslinker that breaks on illumination with ultraviolet (UV) light, triggering a softening of the hydrogel^57^ (Fig. 4a–c). The extent of softening can be regulated by the dose of light. We first verified that the gel softening affected cell mechanotransduction by seeding cells on unexposed gels (Young modulus, ~27 kPa), or gels softened for 4.5 min before cell seeding. As expected, the N/C YAP ratio in cells seeded on the presoftened gels (Fig. 4d,e) was significantly lower. We next utilized photodegradable hydrogels to investigate whether FBs regulate how cells adapt to a change in the mechanical properties of the environment. We seeded cells on non-softened gels for >4 h in the presence of the scramble or PUR4 peptide to control FB formation and observed that in both conditions, YAP was localized to the nucleus (Fig. 4f–h). We subsequently in situ softened the gels with 4.5 min of UV illumination and waited for 1 h to allow the cells to adapt to the new low-rigidity environment. After 1 h, there was a reduction in the N/C YAP ratio for both conditions; however, the cells with FBs (scramble) had a significantly higher N/C YAP ratio compared with the cells lacking FBs (PUR4) (Fig. 4i,j), suggesting that FBs help to maintain a mechanically active phenotype and delay the adaptation timescales. Confirming that this effect is due to substrate mechanics rather than UV illumination, UV exposure to cells on non-degradable polyacrylamide gels of similar rigidity did not produce differences in N/C YAP or cell viability between the two conditions (Extended Data Fig. 8).

**Extended Data Fig. 9:**
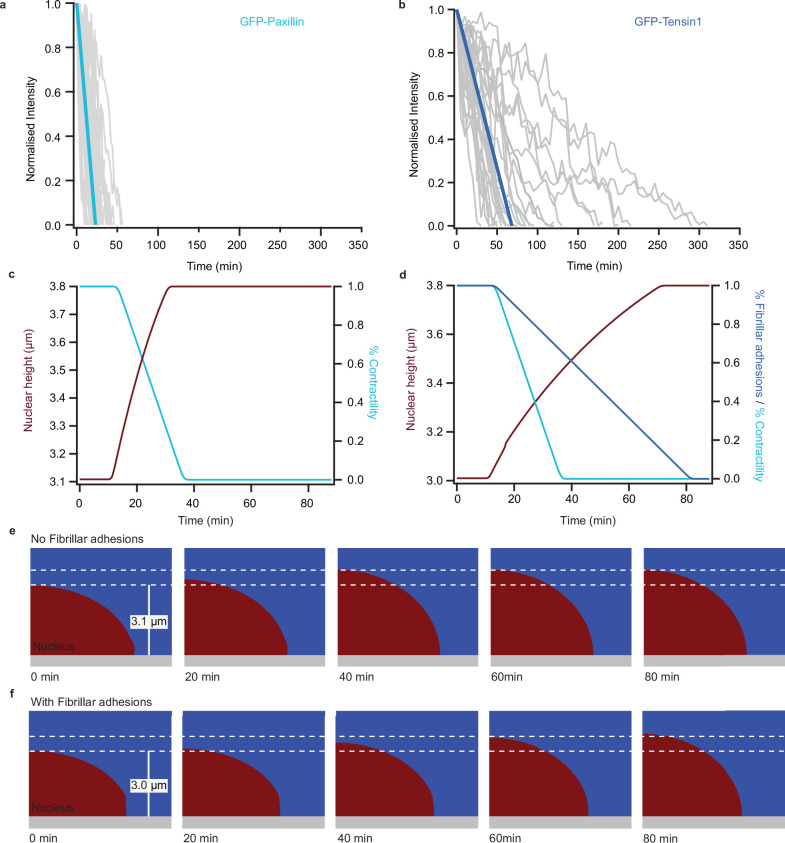
Fibrillar adhesions set the timescales of nuclear remodelling upon loss of contractility. **a**. Normalised intensity curves of the disassembly of focal adhesions (GFP-Paxillin). Individual adhesions (grey) and the average linear decay (cyan). **b**. Normalised intensity curves of the disassembly of FBs (GFP-Tensin1). Individual adhesions (grey) and the average linear decay (blue). **c**. Nuclear height (red) following inhibition of contractility, decaying at the same rate as focal adhesion disassembly (cyan) in the absence of FBs. **d**. Nuclear height (red) following inhibition of contractility, decaying at the same rate as focal adhesion disassembly (cyan) in the presence of FBs, with the associated decay rate (blue). **e**. Snapshots of nuclear height at the indicated times corresponding to the plot in panel **c**. (Corresponds to Supplementary Video 1.). **f**. Snapshots of nuclear height at the indicated times corresponding to the plot in panel **d**. (Corresponds to Supplementary Video 2). Source data

To understand how FBs affect the timescales of mechano-adaptation, we performed in situ softening experiments on cells transfected with Sencyt in the presence of the PUR4 or scrambled peptide (Fig. 4k–o). In the presence of the PUR4 peptide, the N/C ratio of the sensor began to decrease immediately on softening (Fig. 4l). By contrast, control cells with FBs displayed a lag time, where the N/C ratio of the sensor was unaffected by gel softening for ~30 min before decreasing. Correspondingly, the time required for the sensor N/C ratio to fall below half of the starting value (*t*_0.5_) was ~52 min for control cells, compared with ~17 min in the presence of the PUR4 peptide (Fig. 4m). We verified that the initial (presoftened) and final (2 h post-softening) N/C sensor ratio was the same for both conditions (Fig. 4n,o). We, therefore, sought to understand whether these differences in cellular-response timescales stem from differences in dynamics of the focal adhesions compared with the FBs. For cells seeded on both glass and polyacrylamide gels, we analysed the disassembly timescales of focal adhesions (marked with paxillin-GFP; Fig. 4p,q and Extended Data Fig. 8) and FBs (marked with tensin1-GFP; Fig. 4r,s and Extended Data Fig. 8) and found a stark difference. Although the focal adhesions disassembled within ~20 min, FBs required ~75 min (Fig. 4t), thereby closely matching the timescales of adaptation to soft substrates. We then used our computational model to understand if these differences in adhesion dynamics could explain our adaptation timescales. To this end, we performed simulations starting with a contractile cell with a deformed nucleus. Then, we implemented a progressive reduction as a function of time of both actomyosin contractility (assumed to follow the faster decay of focal adhesions) and of slowly decaying FBs, following the measured experimental trends. The simulation recapitulated our experimental findings, where in the presence of FBs, the timescale of nuclear remodelling was governed by the kinetics of adhesion disassembly, whereas in their absence, it was dictated by the faster timescale of contractility inhibition (Extended Data Fig. 9 and Supplementary Videos 1 and 2). Altogether, this demonstrates that the stable dynamics of FBs sets the timescales of cellular mechano-adaptation, thereby delaying the relocalization of mechano-sensitive molecules on softening of the mechanical environment (Fig. 4u).

## ECM–vimentin coupling shields the nucleus from deformation

**Fig. 5:**
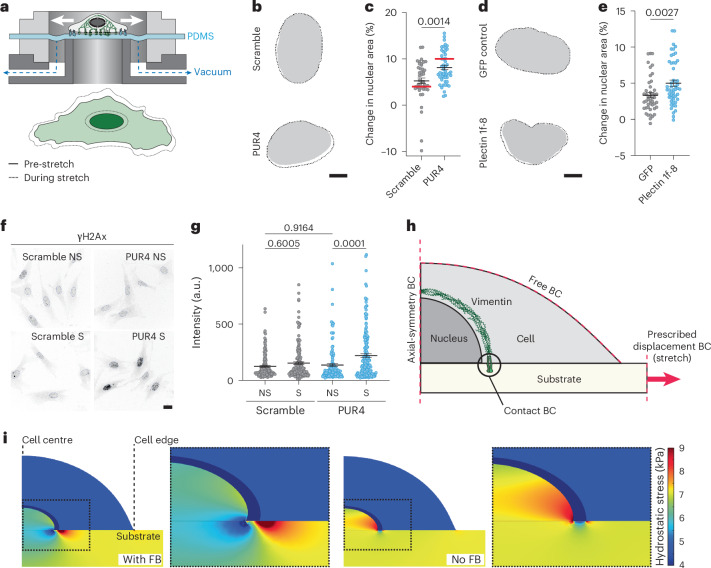
ECM–vimentin coupling protects the nucleus from mechanical deformation and damage. **a**, Schematic of the stretch device. Cell images are acquired before and during the application of a stretch. **b**,**c**, Nuclei before (solid) and during (dashed line) stretch (**b**) and quantification of percentage change in nuclear area on stretch (**c**) for cells in the scramble or PUR4 peptide. Scale bar, 5 µm (*n* = 47/50 cells for scramble/PUR4 from three independent experiments; two-tailed Mann–Whitney test). The red bars correspond to results from the computational model (with/without FBs for scramble/PUR4 conditions). **d**,**e**, Nuclei before (solid) and during (dashed line) stretch (**d**) and quantification of percentage change in nuclear area on stretch (**e**) for GFP- or plectin-1f-8-transfected cells. Scale bar, 5 µm (*n* = 46/51 cells for GFP/plectin 1f-8 from three independent experiments; two-tailed Mann–Whitney test). **f**,**g**, γH2Ax staining (**f**) and quantification of γH2Ax nuclear intensity cells (**g**) in scramble or PUR4 peptide that were not stretched (NS) or subjected to stretch (S). Scale bar, 20 µm (*n* = 132/174/139/175 cells for scramble-NS/scramble-S/PUR4-NS/PUR4-S from two independent experiments; two-way ANOVA with Tukey’s multiple comparisons test). **h**, Schematic of the boundary conditions (BCs; pink dashed lines) and the prescribed substrate displacement (pink arrow) used to simulate the cell-stretching experiments. The contact BC at the vimentin–substrate interface includes a frictional component *η*_d_ when FBs are present (Methods). **i**, Model predictions for stresses in the cell and the substrate during mechanical stretch for cells with an anchored vimentin network (FBs, *η*_d_ = 90 N s µm^−1^) and cells lacking vimentin anchoring (no FBs, *η*_d_ = 0 N s µm^−1^). Data are presented as mean ± s.e.m. Source data

**Extended Data Fig. 10:**
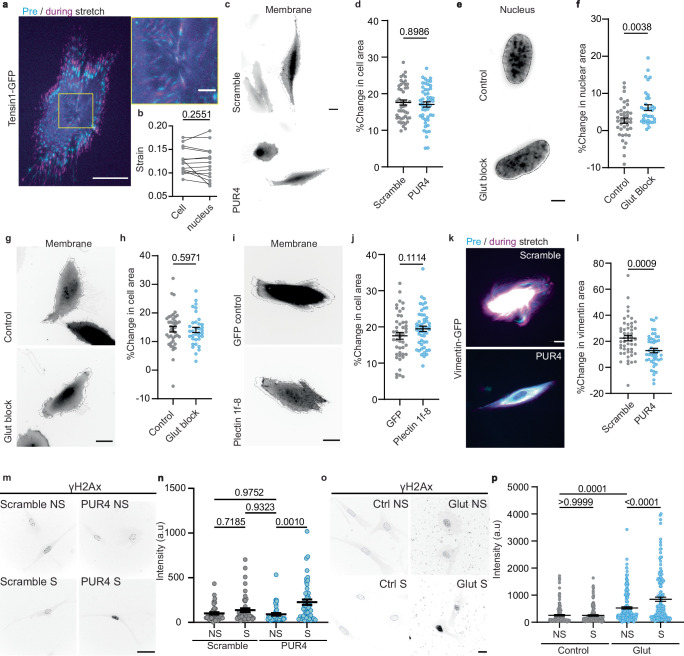
Further characterization of the effect of stretch depending on the existence of FBs. **a**. Tensin1-GFP transfected cell before (cyan) and during (magenta) stretch. Images aligned at an adhesion located centrally under the nucleus. Scale bar is 25 µm / 5 µm zoomed. **b**. Analysis of the whole cell strain and the strain under the nucleus upon a 10% stretch. Two-tailed paired t-test from 3 independent experiments (n = 14 cells). **c,d**. Cell membrane before and during (dashed line) stretch (**c**) and quantification of the percentage change of cell area (**d**) for cells cultured in scrambled or PUR4 peptide. Scale bar 20 µm. (Scramble n = 47, PUR4 n = 51 cells from 3 independent experiments; Two-tailed Mann-Whitney test). **e,f**. Hoechst-stained nuclei before and during (dashed line) stretch (**e**) and percentage change in nuclear area upon stretch (**f**) for cells on control and gluteraldehyde blocked substrates. Scale bar 5 µm. (Control n = 41, cells glut n = 35 cells, from 3 independent experiments; Two-tailed Mann-Whitney test). **g,h**. Cell membrane before and during (dashed line) stretch (**g**) and quantification of the percentage change of cell area (h) for cells on control and gluteraldehyde blocked substrates. Scale bar 20 µm. (control n = 41 cells, glut n = 35 cells from 3 independent experiments; Two-tailed Mann-Whitney test). **i,j**. Cell membrane before and during (dashed line) stretch (**i**) and quantification of the percentage change in cell area (**j**) in cells expressing GFP-only or Plectin 1f-8-GFP. Scale bar 20 µm. (GFP n = 46 cells, Plectin 1f-8 n = 51 cells from 3 independent experiments; Two-tailed Mann-Whitney test). **k,l**. Vimentin-GFP transfected cells before (cyan) and during (magenta) mechanical stretch (**k**) and quantification of percentage change in vimentin area upon stretch for scramble and PUR4 peptide (**l**). Scale bar is 20 µm. (Scramble n = 50 cells, PUR4 n = 47 cells from 3 independent experiments. Two-tailed Mann-Whitney t-test). **m,n**. γ-H2AX staining (**m**) and quantification of γ-H2AX intensity (**n**) in cells lacking cell-cell contacts that were not stretched (NS) or stretched (S) in presence of scramble or PUR4 peptide. Scale bar is 20 µm. (2-way ANOVA from 2 independent experiments, scramble NS n = 43 cells, scramble S n = 51 cells, PUR4 NS n = 43 cells, PUR4 S n = 49 cells). **o,p**. γH2Ax staining (**o**) and quantification of γ-H2AX intensity (**p**) in cells on control or glut blocked substrates that were not stretched (NS) or stretched (S). Scale bar 20 µm. (Control NS n = 156, control S n = 138, glut NS n = 196, glut S n = 140 nuclei from 2 independent experiments; 2-way ANOVA with Tukey’s multiple comparison test). Data are presented as mean +/- SEM. Source data

So far, we have demonstrated that an anchored vimentin network sets the timescales for adaptation on a loss of force. A more well-established role for the intermediate filament network^58,59^, particularly vimentin^49,55^, is that it protects the nucleus from mechanical deformation and damage. However, this knowledge largely stems from studies comparing cells lacking a vimentin network to cells with an intact network. Our results raise the question of whether the vimentin network must merely be present or must be anchored to the ECM to effectively dissipate high mechanical loads. To address this question, we stretched cells by ~10% and measured the corresponding change in cell and nuclear area (Fig. 5a). Cells treated with either PUR4 or the scrambled peptide increased their membrane area equally on stretch (Extended Data Fig. 10). FBs (marked with tensin1-GFP) were also stretched (Extended Data Fig. 10). However, the nuclei of cells with the scrambled peptide (and therefore with FBs) increased their area to a smaller degree than cells exposed to PUR4 (Fig. 5b,c). The same trends were observed when blocking FBs with glutaraldehyde (Extended Data Fig. 10) or with plectin 1f-8 overexpression (Fig. 5d,e and Extended Data Fig. 10). The decreased stretch of nuclei also reduced the well-known effect of stretch-induced DNA damage^12,56,60^, as measured with the marker γH2AX (Fig. 5f,g and Extended Data Fig. 10).

Thus, the vimentin–ECM link mediated by FBs protects the nucleus from external stretch, reducing its deformation. To understand this effect, we carried out simulations in our computational model (Fig. 5h), where we imposed an external 13% stretch to cells (rather than changing the internal cell contractility, as done above). We also assumed that stress between FBs and the substrate is frictional due to the slip-bond nature of adhesions (Methods). This implies that vimentin anchoring to the FBs facilitates energy dissipation to the substrate; indeed, in the condition where vimentin is anchored to the FBs, we observed high stresses in the ECM close to the adhesion sites (Fig. 5i). By contrast, cells with vimentin decoupled from FBs did not dissipate applied stress, leading to increased nuclear deformations. Indeed, predictions of nuclear deformation were lower in cells with FBs (4%) than those without (10%), closely matching the experimental values (Fig. 5c). Taken together, our experimental results in conjunction with a minimal component model reveal that vimentin anchoring to the ECM is crucial for effectively dissipating an applied stretching force and protecting the nucleus from deformation and damage.

## Outlook

Here we demonstrate a mechanism by which matrix remodelling, FB formation and the anchoring of the vimentin network lock the nucleus in a mechanically deformed state. Effectively, this sets a low-pass filter to mechanical stimulation, setting the timescale of nuclear shape changes (and all subsequent mechanotransduction events) to the timescale of FB remodelling (~1 h). This mechanism shows two major new properties of the ECM: (1) it can regulate not only mechano-signalling but its timescale and (2) this regulation depends not only on ECM protein composition^58^ and spatial organization^61^, as reported previously, but also on the conformation of its proteins (in this case, fibronectin).

Our findings also reveal an unanticipated role of vimentin, and of intermediate filaments in general. Intermediate filaments are known to be major determinants of cell mechanical properties^62–65^, on their own and through interactions with the actin cytoskeleton^66–68^. With respect to the nucleus, the mechanical properties of both vimentin^49,55^ and keratin^58,59^ were shown to protect the nucleus from deformation and damage. Here we show that vimentin can have the opposite effect, by maintaining rather than preventing nuclear deformation. This mechanism may also be controlled in different contexts by the rich signalling landscape regulating intermediate filaments, for instance, through phosphorylation^69^ or solubility^70^.

At present, the molecular link between plectin 1f and FBs remains unknown, and multiple molecular binding targets could potentially exist. Tensin is a possible candidate as it is a major component of the FBs^32^, and tensin3 drives FB formation via the interaction with talin^71^. Another candidate is Hic-5, which is fundamental for FB formation and fibronectin remodelling via its interaction with tensin1 (refs. ^72,73^), and its ablation triggers vimentin network collapse^74^. Finally, given that plectin 1f also contains an actin-binding domain close to the N terminus, we cannot exclude the possibility that plectin 1f also interacts with basal actin fibres.

We anticipate that the mechanism unveiled in this work could be relevant in physiological settings in which slow and fast mechanical perturbations must elicit different cellular responses. For instance, fibroblasts in connective tissue associated with lungs, heart, the circulatory system or the urinary system should respond to long-lasting mechanical alterations caused by wounding or tumour formation, but not to second-scale alterations caused by breathing, heart pumping or bladder voiding. Whether such implications remain to be explored, the timescale regulatory mechanism unveiled here sets a foundation to explore such phenomena, with potential implications in homeostasis and disease.

## Methods

### Cell culture, transfections and drug treatments

Human TIFFs (a kind gift from J.I.) were cultured in high-glucose Dulbecco’s modified Eagle’s medium (Thermo Fisher Scientific) supplemented with 20% fetal bovine serum (v/v; Thermo Fisher), 2% of 1-M HEPES (v/v; H0887 Sigma) and 1% penicillin–streptomycin (v/v; 10378016, Thermo Fisher). For stretch experiments, media were changed to CO_2_-independent media (18045054, Thermo Fisher) supplemented with the same concentrations of fetal bovine serum, HEPES and penicillin–streptomycin, as done previously. Mammary epithelial cells (MCF10A) were purchased from ATCC (category number CRL-10317) and cultured in Dulbecco’s modified Eagle’s medium–F12 (21331-020, Life Technologies) with 5% horse serum, 1% penicillin–streptomycin, EGF (20 ng ml^−1^), hydrocortisone (0.5 μg ml^−1^), cholera toxin (100 ng ml^−1^) and insulin (10 μg ml^−1^). All cells were maintained at 37 °C with 5% CO_2_. Cell cultures were routinely checked for the presence of mycoplasma.

Transfections were conducted using the Neon Transfection System (Thermo Fisher Scientific) following the manufacturer’s instructions. TIFF cells were subjected to a single voltage pulse of 1,650 V with a width of 20 ms. Cells were transfected the day before the experiments, and the cells were seeded ~4 h before the experiment, unless otherwise stated.

#### Plasmids used for transfections

The Sencyt mechano-reporter was generated in the laboratory from a previous study^8^. Plectin 1f-GFP and plectin 1f-8-GFP were generated from a previous study^75^. GFP-paxillin was generated in the laboratory from a previous study^3^. Membrane marker *N*-terminal Neuromodulin-GFP was a kind gift from F. Tebar. Tensin1-eGFP was a kind gift from J.I. EGFP-Vimentin-7 was a gift from M. Davidson (Addgene plasmid number 56439; RRID: Addgene_56439).

For the pharmaceutical inhibitor experiments, cells were seeded on fibronectin-coated substrates for a minimum of 4 h (unless otherwise stated) to allow FB formation. All compounds were diluted and stored according to manufacturer’s instructions. Immediately before the experiments, compounds were diluted in cell culture media and warmed to 37 °C before adding to the cells.

The drugs and concentrations used were bleb (25 µM, B0560, Sigma), cytoD (1 µM, C2618, Sigma), Y-27 (25 µM, 688001, Sigma) and latA (0.5 µM, L5163, Sigma). Control cells were incubated with DMSO (Sigma), where the volume added was equal to the maximum volume of the drug conditions.

For the activation of α_5_β_1_ integrin by Mn^2+^, after trypsin cells were resuspended in media containing 5 mM of Mn^2+^ and seeded onto 1.5-kPa polyacrylamide gels. This concentration of Mn^2+^ was maintained throughout the duration of the experiment.

### Fibril-blocking approaches

The PUR4 (also known as FUD) peptide (sequence, KDQSPLAGESGETEYITEVYGNQQNPVDIDKKLPNETGFSGNMVETEDT) and the scrambled control (sequence, EKGYSKPPVGNEGGDQVDEYDTMSQTKLEDEGNTLISPITFENATEQVN) were synthesized by Thermo Fisher Scientific without any tags or modifications. In all the experiments, after trypsin, the cells were resuspended in media containing the PUR4 or scramble peptide at a final concentration of 500 nM. The peptide was maintained in the media for the duration of the experiment.

For blocking α_5_β_1_ integrin, cells in suspension were incubated for 20 min at 37 °C in the blocking antibody (α_5_β_1_ integrin, clone JBS5, Sigma) or control antibody (IgG) before seeding onto 10 µg ml^−1^ of fibronectin-coated glass surfaces. Both blocking antibodies were used at a concentration of 10 µg ml^−1^.

Gluteraldehyde-blocked surfaces were prepared as described previously^47^. Briefly, glass surfaces were coated with 10 μg ml^−1^ of fibronectin overnight at 4 °C or 1 h at 37 °C. Surfaces were then treated with 1% gluteraldehyde (Sigma-Aldrich) in MilliQ H_2_O solution for 10 min at room temperature. Surfaces were then thoroughly rinsed with H_2_O and left to incubate in freshly prepared 1% bovine serum albumin (Sigma-Aldrich) solution for at least 20 min at 37 °C before cell seeding.

### siRNA treatment

siRNA treatment was applied using reverse transfection by incubating detached TIFFs with DharmaFECT 1 Transfection Reagent complexes preincubated with 100 nM of the corresponding siRNA (ON-TARGETplus Non-targeting Pool 20 µM, reference number 77D-001810-10), ON-TARGETplus SMART pool SYNE3 (reference number L-016637-01) and ON-TARGETplus SMART pool VIM (reference number L-003551-00) (all from Dharmacon). The cell suspension with the siRNA complexes was seeded and incubated for 24 h before changing the media, and cells were used after 72 h.

### RNA isolation and quantitative polymerase chain reaction

Total RNA was isolated by using the High Pure RNA Isolation Kit according to the manufacturer’s instructions. Then, 500 ng of RNA was used to generate the corresponding cDNA with the iScript Reverse Transcription Supermix (Bio-Rad). SYBR Green-based quantitative reverse-transcription polymerase chain reactions (Fast SYBR Green Master Mix, Applied Biosystems) were run in triplicate in a StepOnePlus System (Applied Biosystems). The expression level of individual genes was analysed by the ΔCt method and normalized according to the expression of the housekeeping gene *RNA18S*. Primers sequences are listed in Supplementary Table 1.

### Western blotting

Cells were lysed in RIPA buffer (Merck Millipore) with protease and phosphatase inhibitor cocktails (1%; both from Sigma-Aldrich) on ice and then centrifuged at 13,000*g* for 10 min at 4 °C. The protein concentration was determined using the bicinchoninic acid assay method. Here 10 µg of proteins for each sample were loaded in on 4%–20% polyacrylamide gels (Bio-Rad), run at 110 V for 1.5 h and then transferred to a nitrocellulose membrane (GE Healthcare LifeScience) at 30 V overnight. Membranes were blocked with 5% bovine serum albumin in Tris-buffered saline with Tween 20, incubated with diluted primary antibody (anti-nesprin 3, ab186746, Abcam, 1:1,000), anti-vimentin (ab92547, Abcam, 1:2,000), anti-GAPDH (sc-32233, Santa Cruz Biotechnology, 1:1,000) in 5% bovine serum albumin in Tris-buffered saline with Tween 20 at 4 °C overnight, and then probed with the proper secondary horseradish-peroxidase-linked antibody (Jackson ImmunoResearch) at room temperature for 1 h. ImageQuant LAS 4000 mini imaging system (Bio-Rad) was used to detect chemiluminescence.

### Immunostainings

Cells were fixed with 4% paraformaldehyde for 10 min at room temperature and rinsed thrice with phosphate-buffered saline (PBS). Cells were permeabilized with 0.1% Triton X for 5 min and then blocked with 0.5% fish gelatin (Sigma-Aldrich) for 1 h (except manganese-treated cells, which were permeabilized using 0.5% Triton X for 15 min). Cells were incubated with the primary antibody for 1 h, diluted in the 0.5% fish gelatin blocking solution, washed with the blocking solution for 30 min and incubated with the secondary antibody labelled with an Alexa fluorophore (Thermo Fisher, 1:300 dilution) for 1 h. In the case of actin staining, phalloidin (Sigma-Aldrich, 1:1,000) was added with the secondary antibody. Hoechst (1:2,000) was added for 5 min to label the nuclei, and the samples were washed thoroughly.

#### Primary antibodies and their dilutions

YAP (1:300, sc-101199, Santa Cruz Biotechnology) or (1:300, 14074S, Cell Signaling); integrin α5, clone SNAKA51 (1:300, MABT201, Millipore); laminB (1:300 ab16048, Abcam); paxillin (1:300, ab32084, Abcam); twist (1:100, SC-81417, Santa Cruz Biotechnology); snail (1:50, Ab224731, Abcam); tensin1 (1:200, ab233133, Abcam); fibronectin (1:300, F3648, Sigma); vimentin (1:600, ab92547, Abcam); γH2Ax (1:300, 2577, Cell Signaling).

### Polyacrylamide gel

Polyacrylamide gels of variable rigidities were prepared as previously described^8^. Briefly, glass-bottom dishes (MatTek) or glass coverslides were treated with a solution of 3-(trimethoxysilyl)propyl methacrylate (Sigma), acetic acid and 96% ethanol (1:1:14) for a minimum of 10 min. The glass was then thoroughly rinsed in 96% ethanol and dried. Gels were prepared by mixing different concentrations of acrylamide and bis-acrylamide to produce gels of different rigidities according to a previous characterization^8^, with 2% v/v 200-nm-diameter fluorescent carboxylate-modified beads (FluoSpheres, Thermo Fisher Scientific), 0.05% v/v ammonium persulfate (Sigma-Aldrich) and 0.05% tetramethylethylenediamine (Sigma-Aldrich) in PBS 1×. To cast the gels, 22 μl was placed on top of the treated glass and then covered with an 18-mm circular coverslip. Gels were left for 45 min to polymerize at room temperature. Finally, gels were submerged in PBS 1× and the top coverslip was removed. To coat gels, we prepared a mixture containing 10% HEPES (0.5 M, pH 6.0), 0.004% bis-acrylamide, 0.05% Igracure 2959 and 4% acrylic-acid *N*-hydroxysuccinimide/DMSO (10 mg ml^−1^, A8060, Sigma) in MilliQ water. Gels were coated in this mixture and then illuminated with UV light for 8 min. Gels were then washed twice in 50 mM of HEPES at pH 7 and twice in PBS 1×, and incubated with 10 µg ml^−1^ of fibronectin in PBS overnight at 4 °C, sterilized by UV treatment in a laminar-flow hood, washed once with PBS and immediately used.

### Photodegradable compound synthesis

Photodegradable precursors were prepared as previously described^57^. Briefly, the acrylate-functionalized photodegradable monomer was synthesized by suspending 4-[4-(1-hydroxyethyl)-2-methoxy-5-nitrophenoxy]butyric acid (0.0166 mol, Sigma-Aldrich) in anhydrous dichloromethane (90 ml). The mixture was purged with argon; triethylamine (0.0664 mol) was added to the flask by syringe; and acryloyl chloride (0.0547 mol) in dry dichloromethane was added dropwise at 0 °C. The reaction was kept under an argon atmosphere and allowed to proceed overnight at room temperature. The reaction mixture was then added to deionized water (0.5 l) and allowed to stir for 2 h at room temperature, before being extracted with chloroform (5 × 200 ml washes). The organic phase was dried over NaSO_4_ and concentrated by rotary evaporation to obtain the acrylate-functionalized photodegradable crosslinker.

To synthesize the photodegradable PEG crosslinker (PEGdiPDA), the acrylate-functionalized photodegradable monomer (6 mmol) was dissolved in *N*-methyl-2-pyrrolidone (15 ml) and purged with argon. The coupling agent 2-(1H-benzotriazole-1-yl)-1,1,3,3-tetramethyluronium hexafluorophosphate (6.6 mmol), 1-hydroxybenzotriazole (6.6 mmol) and diisopropylethylamine (0.012 mol) were then added to the reaction mixture and stirred for 5 min before the addition of PEGdiamine (0.6 mmol, 2 kDa) in *N*-methyl-2-pyrrolidone. The reaction mixture was heated and vortexed until all the reactants had completely dissolved, and left to stir overnight at room temperature. The reaction mixture was then precipitated in diethyl ether at 0 °C and collected by centrifugation. The macromer product was redissolved in water and centrifuged to yield a dark brown pellet with a clear supernatant. The supernatant was collected, dialysed (SpectraPor 7; CO, 1,000 g mol^−1^) and lyophilized to produce a white powder (39% yield) that was used in experiments.

### Characterization of photodegradable gel mechanical properties

Photodegradable gels were prepared by first mixing 5.4 wt% of PEGdiPDA, 9.6 wt% of PEG400acrylate and 6.6 mM of sodium acrylate in PBS before degassing for 5 min on ice. Polymerization was initiated by the addition of 200-mM tetramethylethylenediamine and 100-mM ammonium persulfate, which were preincubated on ice, and drops were added between Sigmacote (Sigma-Aldrich)-treated glass slides with either 200-µm spacers for 12-µl gels or 100-µm spacers for 6-µl gels. The gels were left to polymerize for 10 min before the top glass slide was removed and the hydrogels were transferred to a well plate with PBS (500 µl). Following equilibration for 30 min in PBS, the hydrogels were transferred to a rheometer (DHR-3, TA Instruments) equipped with a light curing accessory (OmniCure 1000, Lumen Dynamics) and an 8-mm parallel-plate tool to measure the shear properties of the hydrogel. The 6-µl gels were used to track the in situ network evolution during irradiation (365 nm, 10 mW cm^−2^), and the 12-µl gels were used to evaluate the rheological properties of equilibrium swollen samples before and after preselected doses of irradiation. All the rheological characterization experiments utilized a strain of 1% and a frequency of 1 Hz.

### Photodegradable gel cell experiments

Glass-bottom dishes were activated using the same protocol as the polyacrylamide gels. Photodegradable gels were prepared by first mixing 5.4 wt% of PEGdiPDA, 9.6 wt% of PEG400acrylate and 6.6 mM of sodium acrylate in PBS before degassing for 5 min on ice. Polymerization was triggered by the addition of 5% tetramethylethylenediamine and 10% ammonium persulfate (2 M), which were preincubated on ice, and a 22-μl drop of gel mixture was placed in the centre of the glass-bottom dish and covered with an 18-mm coverslip to achieve uniform spreading. The gels were left to polymerize for 10 min before the addition of PBS and the removal of the top coverslip.

For functionalization, we prepared a mixture containing 100 mM of 1-ethyl-3-(3′-dimethylaminopropyl)carbodiimide hydrochloride (8510070025, Sigma) and 200 mM of *N*-hydroxysuccinimide (130672, Sigma) in 20 mM of HEPES buffer at pH 7. Gels were incubated in this mixture for 20 min at 37 °C. The gels were rinsed once with HEPES buffer and once with PBS. The gels were then incubated with 10 µg ml^−1^ of fibronectin overnight at 4 °C. To initiate gel softening, gels were placed under a UV lamp (UVP; 365 nm, 15 W) for 4.5 min. We measured the pH and osmolality of the cell culture media before and after the UV illumination protocol and observed no significant differences.

### Live–dead assay

To quantify the extent of cell death from UV illumination during gel softening experiments, cells were seeded on 15-kPa polyacrylamide gels in either PUR4 or scramble peptide. After 4 h, cells were subjected to a 4.5-min UV illumination and left for 1 h. Cells were then incubated for 10 min in media containing 500 nM of Sytox green nucleic acid stain (Invitrogen) and Hoechst. After washing out the Sytox-containing media, cells were imaged using a ×20 objective and the percentage of Sytox-positive cells was calculated.

### EdU assay

Cell proliferation was assessed using the Click-iT Plus 5-ethynyl-2′-deoxyuridine (EdU) Cell Proliferation Kit (Thermo Fisher Scientific), according to the manufacturer’s protocol. Briefly, cells were seeded onto fibronectin-coated glass-bottom wells and incubated for 4 h in the presence of either scramble or PUR4 peptide. Cells were then treated with either DMSO or cytoD for the indicated duration. EdU was added to the culture medium 1 h before fixation. Cells were fixed with 4% paraformaldehyde in PBS for 10 min at room temperature, permeabilized with 0.5% Triton X-100 and stained using the Click-iT EdU kit reagents followed by Hoechst 33342 to label the nuclei. Samples were imaged using a ×10 objective on an epifluorescence microscope, with each field of view containing approximately 20 cells. The number of EdU-positive cells was quantified per field of view, and the mean across all fields was calculated for each biological replicate.

### Image acquisition

Epifluorescent images were obtained and time-lapse microscopy was performed using an inverted microscope (Nikon ECLIPSE Ti) equipped with thermal, CO_2_ and humidity control. Microscopes were equipped with an ORCA Flash4.0 camera (Hamamatsu) and controlled with MetaMorph (v. 7.7.1.0) or Micro-Manager. Most images were taken with a ×60 objective (Plan Apo; numerical aperture (NA), 1.2; water immersion), unless otherwise stated.

For time-lapse acquisition of the change in mechano-reporter Sencyt localization with drug treatments, a single image was acquired before pharmacological treatment, and then, the images were acquired every 5 min for a total duration of 1 h. For time-lapse acquisition of the change in mechano-reporter Sencyt localization on in situ photodegradable gel softening, images were taken on a Nikon TiE inverted microscope equipped with a spinning-disc confocal unit (Andor) and a Sona scientific complementary metal–oxide–semiconductor camera (Andor), using a ×40 objective (Plan Fluor; NA, 0.75) controlled using Fusion software. A single *z* stack was acquired before UV softening. Gels were then softened for 4.5 min with a UV lamp, and the *z*-stacked images were taken every 10 min for 2 h. For all experiments involving transfected cells, only cells positive for the GFP expression were imaged and analysed.

Confocal images of nuclear height and plectin 1f were acquired a ZEISS LSM880 inverted confocal microscope using ZEISS ZEN2.3 SP1 FP3 (black edition; v. 14.0.24.201) using a ×63, 1.46-NA oil-immersion objective. Confocal images of the vimentin network were taken using a Nikon TiE inverted microscope with a spinning-disc confocal unit (CSU-WD, Yokogawa) and a Zyla 4.2 scientific complementary metal–oxide–semiconductor camera (Andor) using a ×60 objective (Plan Apo; NA, 1.2; water immersion) controlled with Micro-Manager.

### Traction force microscopy

Traction force microscopy experiments were performed as described previously^76^. Briefly, cells were seeded on 15-kPa polyacrylamide gels embedded with fluorescent beads. Images of the cells and the beads were acquired before pharmacological treatment and 30 min after pharmacological treatment. Cells were then removed from the gel using trypsin to obtain a reference image of the beads. Local gel deformation was computed using a custom particle imaging velocimetry software^77^ in MATLAB (MathWorks). Traction forces were computed using Fourier traction microscopy with a finite gel thickness and the mean of each cell was calculated.

### Cell stretch experiments and quantification

Stretchable polydimethylsiloxane (PDMS) (SYLGARD 184 Silicone Elastomer Kit, Dow Corning) membranes were prepared as previously described^9^. Briefly, a base-to-crosslinker mix (10:1) was spun for 1 min at 500 rpm and cured at 65 °C overnight on plastic supports. Once polymerized, membranes were peeled off and assembled onto the stretching device. The PDMS membranes were functionalized with 10 µg ml^−1^ of fibronectin overnight at 4 °C. TIFF cells were seeded for at least 4 h (unless otherwise stated) before the stretch experiment. Immediately before stretch, the cell media were changed to CO_2_-independent media. The stretch experiments were performed by mounting the stretching device on an upright microscope (Nikon ECLIPSE Ni-U) equipped with temperature control and controlled using Metamorph. Calibration of the system was performed using PDMS coated with fluorescent beads, to ensure that the vacuum applied a 10% stretch to the PDMS membrane. Each membrane was stretched for a maximum of six times per experiment. The percentage change in the area of the nucleus and cell membrane on stretch was calculated by segmenting the fluorescent signal from the Hoechst or membrane marker, respectively, before and during stretch. For DNA damage experiments, cells were subjected to five cycles of 30 s of 10% stretch and 10 s of release. The stretch system was immediately removed from the microscope and the cells were fixed and stained with γH2Ax and Hoechst. The Hoechst signal was used to segment the nuclei, and the mean intensity of each nucleus was measured correcting for the background.

### Adhesion disassembly times

To measure the disassembly times of focal adhesions compared with FBs, TIFF cells were transfected with either paxillin-GFP or tensin1-GFP, respectively. Transfections were performed 24 h before the experiment. On the day of the experiment, cells were seeded on fibronectin-coated glass-bottom dishes (MatTek) of polyacrylamide gels and left to form adhesions for a minimum of 4 h. Adhesion dynamics were acquired using a ZEISS LSM880 inverted confocal microscope with a ×63 1.46-NA oil-immersion objective. For cells expressing paxillin-GFP, images were acquired every 120 s for approximately 2.5 h. For cells expressing tensin1-GFP, images were acquired every 300 s for approximately 10 h. The adhesion intensity was tracked with time, from the initial formation until disappearance. The plot of adhesion intensity was then fit with a Gaussian, and the disassembly time was measured at the time from the Gaussian peak until the return to background levels.

### Micropatterning experiments

Single rectangular adhesive patterns were generated on glass substrates using PRIMO2 UV light patterning system (Alvéole) mounted on an inverted microscope (Nikon ECLIPSE Ti2). Glass coverslips were prepared for patterning by 1-min plasma cleaning followed by immediate incubation with poly-L-lysine for 30 min. The glass slides were profusely washed with 0.1-M HEPES buffer (pH 8.5), and incubated for 1 h in 70 mg ml^−1^ of PEG-SVA (*M*_w_, 5,000; Laysan Bio). Glass coverslips were then washed in MilliQ water and left to dry in a laminar-flow hood. Finally, PLPP gel (Alvéole), diluted at 1:10 in 70% ethanol, was placed on the coverslips and allowed to dry completely. After UV patterning, coverslips were washed thoroughly and then incubated for 5 min with fibronectin (0.1 mg ml^−1^) and fibrinogen-647 (0.01 mg ml^−1^) to facilitate pattern visualization.

### Image analysis

#### N/C ratio analysis

The N/C ratio was quantified by measuring the mean fluorescence intensity of a nuclear region (*I*_nucleus_) and the intensity of a cytoplasmic region immediately adjacent to it (*I*_cytoplasm_). The nuclear region was determined from the Hoechst staining. The ratio was calculated using the following formula:

1 $$\frac{{\rm{Nucleus}}}{{\rm{Cytoplasm}}}=\frac{{I}_{{\rm{nucleus}}}-{I}_{{\rm{background}}}}{{I}_{{\rm{cytoplasm}}}-{I}_{{\rm{background}}}},$$

where *I*_background_ is the mean fluorescence intensity of a region outside of the cell. For the quantification of the mechano-reporter Sencyt with time, the N/C ratio was calculated at each time point. For all drug treatment experiments, the N/C ratio at each time point was normalized to the N/C ratio before the addition of the compound. For quantification of the N/C mechano-reporter Sencyt ratio during in situ gel softening experiments, a single confocal plane was selected and the N/C ratio was normalized between the presoftened N/C ratio and the ratio at the final time point.

#### FB quantification

FBs were marked with integrin α5, clone SNAKA51 antibody (or tensin1 antibody for the blocking antibody experiments). To quantify the extent of FB formation, the fibrils in the area under the nucleus (determined from Hoechst staining) were detected using the Fiji Ridge Detection plug-in, and the percentage area occupied by the fibrils was computed. For cells seeded on soft gels, the length of the FBs was calculated to circumvent changes in the focal plane across the whole cell. For each cell, the length of almost five representative FBs under the nucleus were measured.

#### Focal adhesion length

Focal adhesion length was obtained by measuring the length of almost five representative focal adhesions at the cell periphery for each cell.

#### Nuclear height

Nuclear height was measured from *z*-stacked confocal images of laminB-stained nuclei. Each nucleus was resliced along the long axis, an intensity profile was created and the height was measured from the distance between the two peaks of maximum laminB intensity.

#### Vimentin spreading

To calculate the area occupied by vimentin, confocal stacks were acquired for cells stained with actin and vimentin. The area of the actin and vimentin networks was calculated by thresholding the *z* projection (sum) of each channel. The percentage area of the vimentin network with respect to the total cell area (from the actin network) was computed for each cell.

#### Actin anisotropy

The actin anisotropy was analysed using the FibrilTool plug-in in ImageJ^78^.

### Computational model

#### Scope and limitations of the model

The scope of the computational model presented here is that of cellular and nuclear mechanics. It has been designed to capture the essential mechanical interactions governing nuclear deformation and adaptation timescales. It seeks to understand whether the role of FBs as anchors between the intermediate filament cytoskeleton and the substrate can explain how FBs regulate nuclear shape in response to the two types of mechanical stimulus applied experimentally: actomyosin contractility inhibition and cell stretch. In this regard, it does not address aspects upstream (such as the molecular regulation leading to different timescales for focal adhesions and FBs) or downstream (such as how nuclear deformation regulates YAP nucleocytoplasmic transport).

The model is deterministic, with flexibility of the parameter space, which can be adjusted to reflect different cell types, substrate rigidities or pharmacological perturbations, thereby enabling the exploration of qualitative trends across a range of conditions. The model is not intended to provide exact quantitative predictions for all cellular contexts, but rather to highlight the relative contributions of cytoskeletal contractility, vimentin anchoring and FB stability to nuclear mechanics. Limitations include the absence of stochasticity in adhesion bond dynamics, the assumption of isotropy within cytoskeletal elements and the treatment of the nucleus as a linearly elastic material, without explicitly incorporating potential viscoelastic relaxation timescales. Although such viscoelastic effects could shift the magnitude of nuclear deformations, they would not alter the qualitative conclusion that FB anchoring regulates the persistence of nuclear mechanotransduction.

#### Constitutive models for the cell, nucleus and substrate

To fully describe the effect of mechanical stress (generated due to cellular contraction and/or applied stretch) on the nucleus, we consider the following key components in our computational model: (1) contraction due to myosin motors (Fig. 3m, red), (2) actin filaments (Fig. 3m, blue), (3) VIFs (Fig. 3m, green), (4) microtubules (Supplementary Fig. 3, brown) and (5) FBs. In our model, the cell cytoskeleton is considered to consist of spatially varying representative volume elements, each of which comprises components (1)–(4) described above (Supplementary Fig. 3a). We initially assume uniform and isotropic distribution of these elements and describe how—due to the action of contractile forces and the resulting stress field—these cytoskeletal components are redistributed in a more anisotropic manner, facilitating force transfer from the cell cytoskeleton to the nucleus. Also, the ECM is modelled as a linear elastic material with an elastic modulus of 70 kPa, whereas the nucleus is similarly modelled as an elastic material with the Young modulus and shear modulus values listed in Supplementary Table 2. We describe each of these components here.

#### Cytoskeletal contraction due to myosin molecular motors

Myosin motors are treated as force dipoles (pair of equal but oppositely oriented forces) that bind to actin filaments and generate cellular contractility^79^ (Fig. 3m). The volume-averaged density of the bound motors can be represented by a symmetric tensor ${\rho }_{{ij}}$, whose components represent cytoskeletal contractility along different directions^80^. Within our coarse-grained approach, the contraction due to myosin motors is represented as an isotropic stress tensor (*ρ*_11_ = *ρ*_22_ = *ρ*_33_ = *ρ*) with a magnitude of 1.5 kPa, applied at every point in the cell cytoskeleton. Due to cytoskeletal contraction, compressive stress ${C}_{{ijkl}}^{\,({\rm{A}})}{\varepsilon }_{{kl}}^{({\rm{A}})}$ are generated in components in compression (like vimentin), whereas tensile stresses ${s}_{{ij}}$ are generated in the cytoskeletal components under tensile strain (actin filaments). By force balance, the contractility is given as

2 $${\rho }_{{ij}}=-{C}_{{ijkl}}^{\,\left({\rm{A}}\right)}{\varepsilon }_{{kl}}^{\left({\rm{A}}\right)}+{s}_{{ij}},$$

where ${C}_{{ijkl}}^{\,\left({\rm{A}}\right)}$ and ${\varepsilon }_{{kl}}^{\left({\rm{A}}\right)}$ are the stiffness tensor and strain of the components in compression (Supplementary Fig. 3), respectively, namely, the microtubules and vimentin.

#### Actin filaments and actin–vimentin interactions

The actin filaments experience tension as the cell contracts and, hence, are in series with the myosin element. VIFs interact with actin through direct physical contact facilitated by crosslinkers and direct binding^81^. Hence, the VIFs in contact with actin also experience tensile stresses and are added in series to the nonlinear elastic element representing actin filaments (Supplementary Fig. 3).

#### Vimentin–microtubule network under compression

VIFs near the perinuclear region interact with microtubule elements and are experimentally reported to stabilize them^82,83^. To represent this effect, we add another set of vimentin elements in parallel with the microtubules in compression. Hence, there are two sets of VIFs, one that is in direct physical contact with actin and under tension, whereas the other is enmeshed with microtubules under compression, reinforcing them^67^. Also, VIFs have been observed to stiffen under compressive strains, leading to an overall compressive stiffening of cytoskeletal networks^55^. To represent the above effects, the cytoskeleton is modelled as a nearly incompressible, hyperelastic solid that stiffens under compressive strains. First, we define the deformation gradient ${F}_{{ij}}=\frac{\partial {x}_{i}}{\partial {X}_{j}}$ as a second-order tensor that maps infinitesimal line elements ${\rm{d}}{\bf{X}}$ in the reference configuration to corresponding infinitesimal line elements ${\rm{d}}{\bf{x}}$ in the current configuration. Further, we define $C={F}^{{\rm{T}}}F$ to be the right Cauchy–Green deformation tensor whose normal components represent stretch along a given direction, whereas shear components represent a change in angle. A Mooney–Rivlin constitutive equation is used to represent this stiffening behaviour and the strain energy of the cell can be defined as

3 $${W}_{{\rm{s}}}={C}_{1}\left({\bar{I}}_{1}-3\right)+{C}_{2}\left({\bar{I}}_{2}-3\right)+{\frac{1}{2}K\left(J-1\right)}^{\!2}.$$

In the above equation, the Jacobian $J=\det \left({\bf{F}}\right)$ is the determinant of the deformation gradient tensor, whereas ${\bar{I}}_{1}$ and ${\bar{I}}_{2}$ are the first and second invariants of the deviatoric part of ${\bf{C}}$, respectively. The parameters *C*_1_ and *C*_2_ are Mooney–Rivlin parameters, whereas $K$ is the bulk modulus of the cell. In the limit of small strains, the parameters ${C}_{1}$ and ${C}_{2}$ can be related to the shear modulus of the cell $\mu$ as $\mu =2({C}_{1}+{C}_{2})$. The values of these parameters are listed in Supplementary Table 2, and the elastic modulus of the cell for compressive strains in the range of 0.001 to 0.5 is found to be in the range of 2.1–2.9 kPa, which provides reasonable agreement with the Young modulus measured for living fibroblasts using atomic force microscopy^84^. The high levels of compressive strain near the nucleus due to the contractile stress leads to the formation of a stiff region representing the vimentin cage observed experimentally (principal stress ${s}_{3}$; Supplementary Fig. 3). By contrast, in the cell cytoskeleton, high tensile stresses are observed close to the basal plane, particularly near the cell periphery (principal stress ${s}_{1}$; Supplementary Fig. 3), representing the actin and VIFs in tension.

#### Adhesive and frictional forces due to FBs

Due to cytoskeletal contraction, the vimentin cage around the nucleus is gradually pushed down and is anchored by FBs that are present near the centre of the cell. On loss of contractile forces, the nucleus tries to rebound and restore a rounded shape, but this is prevented by the anchored vimentin network. This leads to the application of force from the nucleus to the vimentin network, which is transmitted to the substrate through FBs. We model this as a vertical resistive adhesive force *f*_a_ mediated by FBs on the edge of the vimentin cage, which remains even when contractile forces are removed. We estimate this force to be of the same magnitude as the contractile force needed to push down the vimentin cage.

FBs are defined by α_5_β_1_ integrin and tensin family of proteins, which form bonds with ligands on the ECM. On the application of stretch, the bond between the FBs and the ligands on the ECM (a polymer structure) is ruptured. This rupture corresponds to overcoming energy barriers by thermal activation (note that we assume that the vimentin remains anchored to the FBs as it slides). The disengagement of the FB–ligand bond follows a Hill-type relation, where the velocity of the sliding adhesions decays exponentially as the activation energy associated with bond rupture ${E}_{{\rm{A}}}$ increases:

4 $${v}_{{\rm{a}}}={v}_{0}\exp (-{E}_{{\rm{A}}}/{E}_{0}),$$

where ${v}_{0}$ is the maximum sliding velocity (when the activation energy ${E}_{{\rm{A}}}$ is zero) and ${E}_{0}$ is an energy scale related to thermal or active noise. The activation energy can be expressed as the work done by a dissipative force ${f}_{{\rm{d}}}$ in translocating the FBs by a molecular sliding distance ${\rm{A}}$:

5 $${v}_{{\rm{a}}}={v}_{0}\exp (-{f}_{{\rm{d}}}{\rm{a}}/{E}_{0}).$$

Assuming that the activation energy associated with the rupture is much smaller than ${E}_{0}$, the above equation can be linearized, and expressed as

6 $${f}_{{\rm{d}}}={\eta }_{{\rm{d}}}{v}_{{\rm{a}}},$$

where ${\eta }_{{\rm{d}}}={E}_{0}/{\rm{a}}{v}_{0}$ is a frictional dissipative constant that is inversely related to the mobility of individual FBs^85^. Setting *η*_d_ as zero is equivalent to the case of FBs not anchored to the ECM and is representative of cells lacking FBs.

#### Model sensitivity analysis

For the different parameters, sensitivity analysis was performed within COMSOL Multiphysics using the built-in sensitivity study node. Each parameter of interest (bulk and shear moduli of the nucleus, cell elastic modulus, Mooney–Rivlin parameters and myosin contractile stress) was defined in the ‘Global Parameters’ node of model builder in COMSOL and systematically perturbed around its baseline value. COMSOL computes sensitivities by differentiating the model equations with respect to each parameter and reports the normalized change in selected output variables. In this case, the primary read-out was nuclear deformation, which served as a proxy for mechanotransduction. The resulting sensitivity coefficients quantify the relative influence of each parameter on nuclear mechanics: negative values indicate that increasing the parameter reduces nuclear deformation, whereas positive values indicate that increasing the parameter amplifies nuclear deformation. This analysis allowed us to identify which mechanical properties and active stresses most strongly control nuclear behaviour in the model. As expected, parameters quantifying stiffness (of the cell and nucleus) reduce nuclear deformations, whereas the contractility parameter increases it, with sensitivities of the same order (Supplementary Table 3).

#### Time-dependent implementation of adhesion disassembly

To incorporate the different disassembly timescales of focal adhesions and FBs, the corresponding parameters in the COMSOL model were defined as time-dependent functions. Specifically, contractility and adhesion anchoring strengths were assigned linear decay functions in the ‘Global Definitions→Functions’ node, where the slope was set by the experimentally observed rate of disassembly and the function terminated at the experimentally measured final time (20 min for focal adhesions and ~80 min for FBs). Simulations were carried out in a time-dependent study node using the default backward differentiation formula implicit solver, with the total simulated duration set to 100 min to capture the full relaxation process. Nuclear height was extracted as a function of time, providing a direct read-out of how adhesion-dependent anchoring regulated the kinetics of nuclear rebound following loss of contractility.

#### Geometry, mesh and boundary conditions

The model for the cell cytoskeleton, nucleus and FBs is implemented in COMSOL Multiphysics, within a finite element framework. The cell is modelled as an ellipsoid with semi-axes lengths of 15 and 12 µm, whereas the nucleus is modelled as a spheroid with a radius of 3.7 µm located at the centre of the cell. The substrate is modelled as a rigid cylinder with a radius of 50 µm. Due to rotational symmetry of the cell–substrate system, an axisymmetric analysis is conducted, with horizontal roller boundary conditions applied to the top and bottom ends of the substrate. Although the cell and nucleus fully rest on the substrate, a small hemispherical region around the 0.5-µm-thick nucleus and separated from the nucleus by 0.1 µm is initially separated by a gap of 0.03 µm from the substrate. This represents the vimentin cage that forms on the action of contractile forces and is pushed down and eventually is anchored to the FBs. Standard contact conditions are implemented at the vimentin–nucleus interface, representing the effect of nesprins and other molecules that directly transfer contractile stresses to the nucleus. In addition, the contact boundary conditions are implemented at the vimentin–substrate interface to represent adhesion between the vimentin and FBs near the centre of the cell, using a spring foundation boundary condition with an added friction node. When combined with the frictional contact formulation, this provides a dissipative resistance to tangential motion, capturing the slip–stick behaviour of FBs. In the absence of FBs, the friction node is disabled, whereas the elastic component of the spring foundation is retained, effectively modelling basal substrate contacts and removing the stabilizing contribution of FBs. Triangular mesh elements are used to discretize the cell geometry with a minimum element size of 0.001 µm near the contact zones to accurately resolve the stresses and displacements at contact.

#### Implementation of simulations

To run the simulations, we first defined the axisymmetric geometry of the cell, nucleus and substrate, and then assigned the parameters listed in Supplementary Table 2 together with the boundary conditions described above. Two types of experimental condition were reproduced. In the first, contractility (*ρ*) and FB strength were reduced either as a steady-state change or as a time-dependent decay. In the second, substrate stretch was applied through a prescribed displacement using a ramp function. Both conditions were simulated with FBs present and without FBs by setting the FB-related resistive adhesion force (${f}_{{\rm{A}}}$) and the frictional viscosity constant (${\eta }_{{\rm{d}}}$) to zero. The primary outputs were nuclear height, deformation fields, stress maps and parameter sensitivities.

### Statistics and reproducibility

No statistical method was used to predetermine the sample size. No data were excluded from the analyses. Statistical analyses were performed using GraphPad Prism software (v. 9). The names of the statistical tests used and the number of data points and independent replicates are detailed in the figure captions.

### Reporting summary

Further information on research design is available in the Nature Portfolio Reporting Summary linked to this article.

## Online content

Any methods, additional references, Nature Portfolio reporting summaries, source data, extended data, supplementary information, acknowledgements, peer review information; details of author contributions and competing interests; and statements of data and code availability are available at 10.1038/s41563-026-02590-x.

## Supplementary information

Supplementary Information Supplementary Figs. 1–3, Tables 1–3, captions for Supplementary Videos 1 and 2 and references.

Reporting Summary

Supplementary Video Computational simulation of nuclear height dynamics following the loss of contractility in the absence of FBs. Time-dependent change in nuclear height on the cessation of actomyosin contractility in the absence of FBs (Extended Data Fig. 9c,e).

Supplementary Video Computational simulation of nuclear height dynamics following the loss of contractility in the presence of FBs. Time-dependent change in nuclear height on the cessation of actomyosin contractility in the presence of FBs (Extended Data Fig. 9d,f).

Supplementary Data Statistical source data for the data presented in supplementary figures.

## Source data

Source Data Figs. 1–5 Statistical source data for all data presented in Figs. 1–5 (data of each figure in a separate sheet).

Source Data Extended Data Figs. 1–10 Statistical source data for all data presented in Extended Data Figs. 1–10 (data of each figure in a separate sheet).

Source Data Extended Data Figs. 5 and 6 Unprocessed western blots for Extended Data Figs. 5g and 6e.

## Data Availability

The data supporting the findings of this study are available within the article and its Supplementary Information. Source data are available at 10.34810/data2992. Source data are provided with this paper.

## Code availability

All simulations were performed in COMSOL Multiphysics. The full set of model files, including the COMSOL simulation models and sensitivity analysis model, is available via GitHub at https://github.com/vs-vivek/Nuclear_Mechano_Response and via Zenodo at 10.5281/zenodo.19238182 (ref. ^86^). The repository provides detailed instructions for setup and execution, including example files and guidance on reproducing the results shown in this study. Running the models requires COMSOL Multiphysics (v. 6.2) or later with the Structural Mechanics module. Standard system requirements for COMSOL (multi-core CPU, 16-GB RAM or higher are recommended) are sufficient to execute the simulations.
