# Supplementary material for: Fibrillar adhesion dynamics govern the timescales of nuclear mechano-response via the vimentin cytoskeleton
Source: Nat Mater. 2026 Apr 29;25(7):1252–63. doi: 10.1038/s41563-026-02590-x (PMC13322966; doi:10.1038/s41563-026-02590-x)
Supplement: Supplementary file 1 — Supplementary Figs. 1–3, Tables 1–3, captions for Supplementary Videos 1 and 2 and references. [file 41563_2026_2590_MOESM1_ESM.pdf]

nature materials

Supplementary information

https://doi.org/10.1038/s41563-026-02590-x

Fibrillar adhesion dynamics govern the timescales of nuclear mechano-response via the vimentin cytoskeleton

In the format provided by the authors and unedited

Supplementary Information

Table of Contents:

**Supplementary Video captions**	2
**Supplementary Figure 1.**	3-4
**Supplementary Figure 2.**	5-6
**Supplementary Figure 3.**	7
**Supplementary Table 1.** Primer sequences used.	8
**Supplementary Table 2.** List of parameters used in the simulations.	9
**Supplementary Table 3.** Sensitivity analysis of model parameters.	9
**Supplementary References**	10

Supplementary Video captions:

Supplementary Video 1: Computational simulation of nuclear height dynamics following loss of contractility in the absence of FBs. The time-dependent change in nuclear height upon the cessation of actomyosin contractility in the absence of FBs. Related to Extended Data Figure 9 c and e.

Supplementary Video 2: Computational simulation of nuclear height dynamics following loss of contractility in the presence of FBs. The time-dependent change in nuclear height upon the cessation of actomyosin contractility in the presence of FBs. Related to Extended Data Figure 9 d and f.

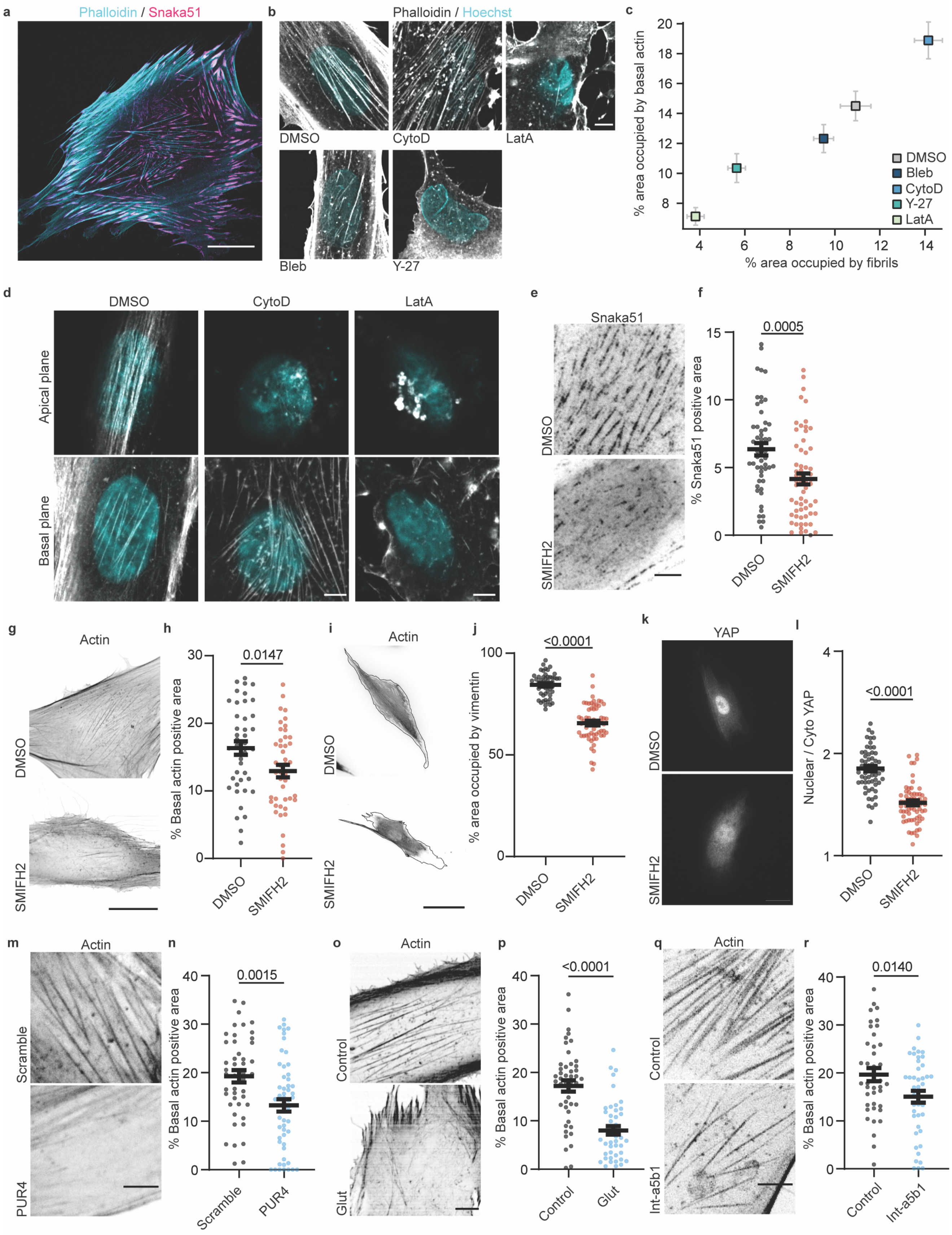
a
Phalloidin / Snaka51
b
Phalloidin / Hoechst
DMSO
CytoD
LatA
Bleb
Y-27
c
% area occupied by basal actin
% area occupied by fibrils
DMSO
Bleb
CytoD
Y-27
LatA
d
DMSO
CytoD
LatA
Apical plane
Basal plane
e
Snaka51
DMSO
SMIFH2
f
0.0005
% Snaka51 positive area
DMSO
SMIFH2
g
Actin
DMSO
SMIFH2
h
0.0147
% Basal actin positive area
DMSO
SMIFH2
i
Actin
DMSO
SMIFH2
j
<0.0001
% area occupied by vimentin
DMSO
SMIFH2
k
YAP
DMSO
SMIFH2
l
<0.0001
Nuclear / Cyto YAP
DMSO
SMIFH2
m
Actin
Scramble
PUR4
n
0.0015
% Basal actin positive area
Scramble
PUR4
o
Actin
Control
Glut
p
<0.0001
% Basal actin positive area
Control
Glut
q
Actin
Control
Int-a5b1
r
0.0140
% Basal actin positive area
Control
Int-a5b1

Supplementary Figure 1. The loss of fibrillar adhesions coincides with the loss of basal actin stress fibres underneath the nucleus. a. Example image of the basal plane of a TIFF cell stained for actin (phalloidin, cyan) and fibrillar adhesions (integrin-α5, clone snaka51, magenta). Image is a confocal slide of the basal plane of the cell; scale bar 25 µm. **b.** Example confocal images (single slice) of actin (white) on the basal plane of the cell situated underneath the nucleus (cyan) upon different pharmacological treatments. Scale bar 5 µm. **c.** Corresponding quantifications. Upon pharmacological treatment, the percentage area occupied by fibrillar adhesions under the nucleus scales with the percentage area occupied by basal actin fibres (For basal actin; DMSO n=48 cells, bleb n=45, cytoD n=46, Y-27 n=45, latA n=51. For fibrillar adhesions; DMSO n=52 cells, bleb n=46, cytoD n=48, Y-27 n=45, latA n=76. Data from at least 3 independent experiments). **d.** Example confocal slice images of the nucleus (cyan) and actin (white) on the apical and basal plane of the cell in cells treated with DMSO, cytoD or latA. Scale bar is 5 µm. **e.** Example images of integrin-α5, clone snaka51 stained cells after 30 minute treatment with DMSO or SMIFH2. Scale bar 5 µm. **f.** Percentage area under the nucleus occupied by fibrillar adhesions in DMSO or SMIFH2 (DMSO n=52 cells, SMIFH2 n=61 cells from 3 independent experiments. Two-tailed unpaired t-test.) **g.** Example images (single confocal slice) of actin on the basal plane of cells after 30 minute treatment with DMSO or SMIFH2. Scale bar 25 µm. **h.** Percentage area under the nucleus occupied by basal actin fibres in DMSO or SMIFH2 (DMSO n=42 cells, SMIFH2 n=45 cells from 3 independent experiments. Two-tailed unpaired t-test.) **i.** Example images of vimentin stained cells treated for 30 minutes with DMSO or SMIFH2. The solid black line indicates the periphery of the cell from phalloidin staining. Scale bar is 50 µm. **j.** Percentage area of the cell occupied by vimentin after 30 minute treatment with DMSO or SMIFH2. (DMSO n=51 cells, SMIFH2 n=62 cells from 3 independent experiments. Two-tailed unpaired t-test). **k.** Example images of YAP stained cells treated for 30 minutes with DMSO or SMIFH2. Scale bar 25 µm. **l.** Nuclear / cytoplasmic ratio for cells treated with DMSO and SMIFH2. (DMSO n=60, SMIFH2 n=66 cells, from 3 independent experiments. Two-tailed Mann-Whitney test). **m,n.** Basal actin in scrambled peptide or PUR peptide (scramble n=45 cells, PUR4 n=53 cells from 4 independent experiments. Unpaired t-test). **o,p.** Basal actin in control or gluteraldehyde treated surfaces (Control n=46 cells, glut n=45 cells from 4 independent experiments. Two-tailed Mann-Whitney test). **q,r.** Basal actin in control blocking antibody or integrin $\alpha_5\beta_1$ antibody (Control n=40 cells, integrin α5β1 n=44 cells from 3 independent experiments; Two-tailed Mann-Whitney test). Scale bars are 5 µm. All data are presented as mean values +/- SEM.

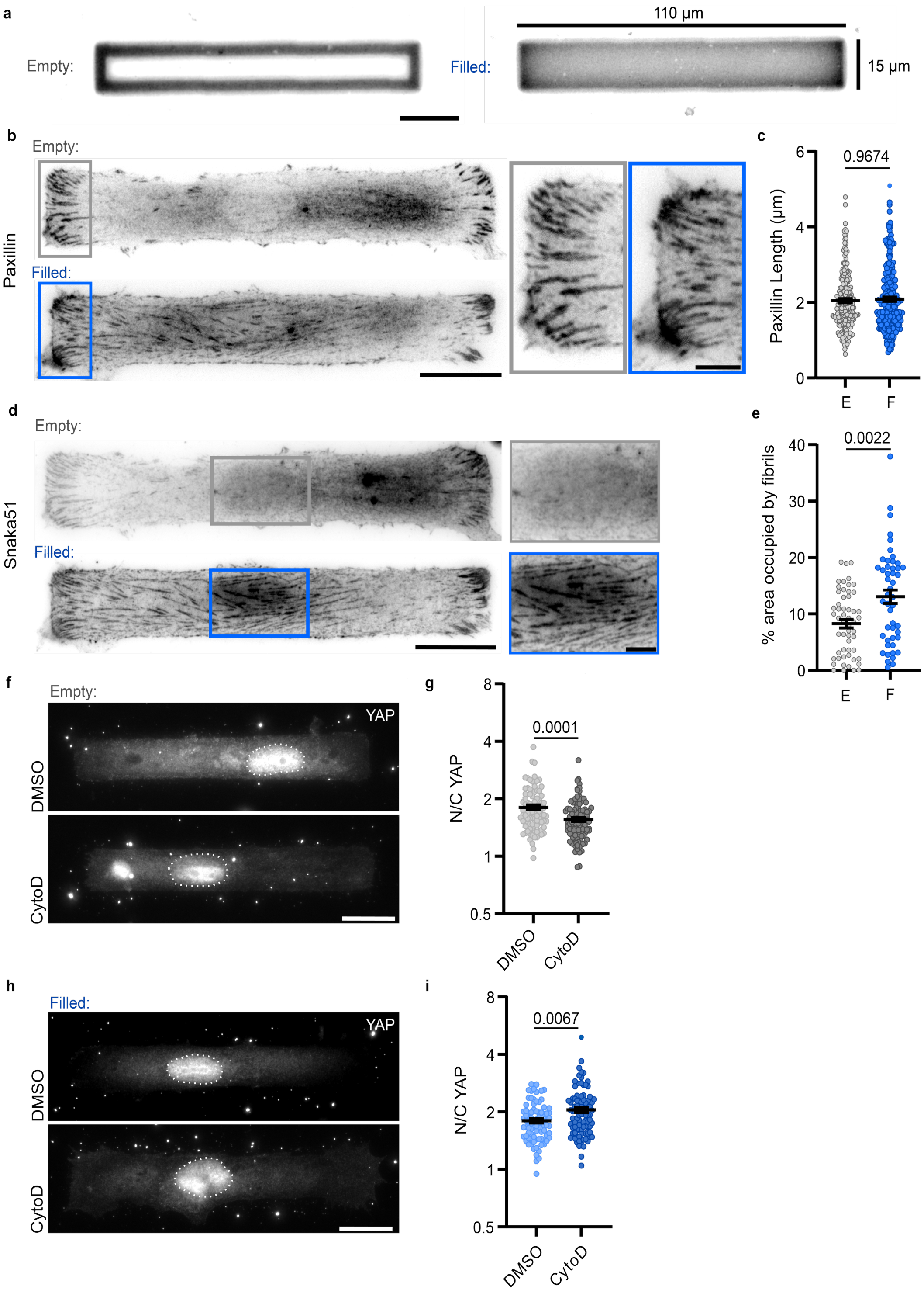

Supplementary Figure 2. Inhibition of fibrillar adhesions via ECM micropatterning leads to loss of nuclear YAP upon loss of contractility. a. Images of the empty and filled micropatterns. In the empty patterns, the line thickness is 4 μm. **b.** Images of cells on empty and filled patterns stained with paxillin. Scale bar 20 μm / zoom scale bar 5 μm. **c.** Analysis of focal adhesion cell for cells on empty (E) and filled (F) patterns. (Empty n=210 adhesions from

42 cells, Filled n=219 adhesions from 44 cells, from 3 independent experiments; Two-tailed Mann-Whitney test). **d.** Images of cells on patterns stained for fibrillar adhesions using the snaka51 antibody. Scale bar 20 μm / zoom scale bar 5 μm. **e.** Analysis of the percentage area occupied by fibrillar adhesions below the nucleus for cells on empty and filled patterns (Empty n=55 cells, filled n=48 cells from 3 independent experiments; Two-tailed Mann-Whitney). **f.** Images of YAP in cells on empty patterns treated with DMSO or cytoD. Dashed white line represents nuclear periphery. Scale bar is 20 μm. **g.** Analysis of N/C YAP for DMSO and cytoD treatment of cells on empty patterns. (DMSO n=90 cells, cytoD n=110 cells from 3 independent experiments; Two-tailed Mann-Whitney). **h.** Images of YAP in cells on filled patterns treated with DMSO or cytoD. Dashed white line represents nuclear periphery. Scale bar is 20 μm. **i.** Analysis of N/C YAP for DMSO and cytoD treatment of cells on filled patterns. (DMSO n=84 cells, cytoD n=92 cells from 3 independent experiments; Two-tailed Mann-Whitney). All data are presented as mean values +/- SEM.

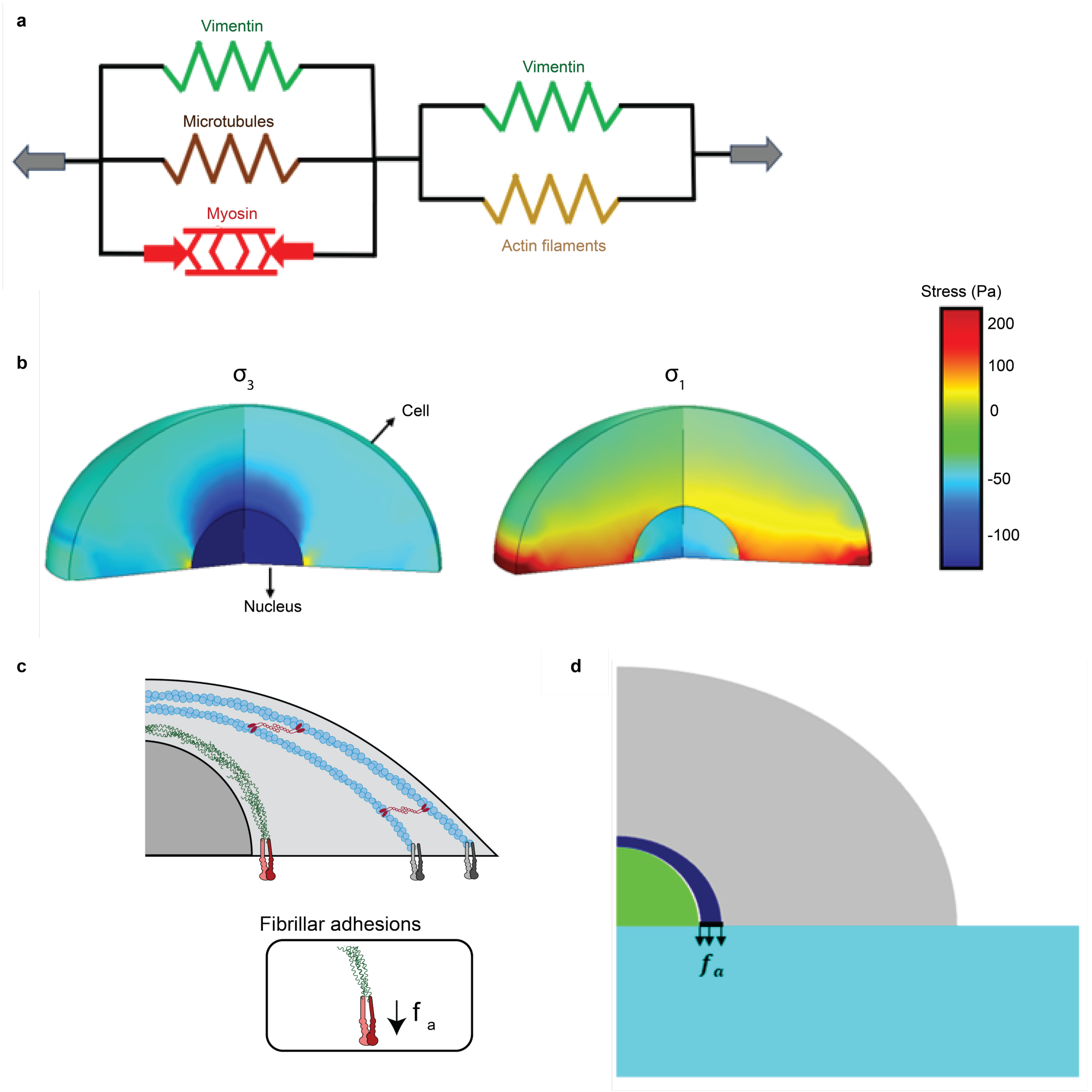

Supplementary Figure 3. Additional characterisation of the computationally modelling methods. a. A one-dimensional representation of the chief components in the computational model. Vimentin intermediate filaments in direct physical contact with actin are in tension and essential for force transmission to the ECM while VIFs adjacent to the nucleus reinforce the microtubules and are under compression. **b.** Principal Stress distribution within the cell due to contractile forces, where $\sigma_1>\sigma_2>\sigma_3$. Maximum compressive stress field is found around the nucleus and represents the formation of vimentin cage, while the maximum tensile stress is observed along the basal plane and close to the cell periphery. **c.** Schematic representation of the engagement of vimentin cage with fibrillar adhesions due to cell contraction. **d.** Schematic of model geometry describing how the adhesive forces are applied to represent anchoring by adhesions.

Supplementary Table 1. Primer sequences used.

hSYNE3-F	GCAGGTGACAAGTTCTGTGAGG
hSYNE3-R	TGGAGGTCTAGGAGCTTCCTGT
hVIM-F	GCAAAGATTCCACTTTGCGT
hVIM-R	GAAATTGCAGGAGGAGATGC
hRNA18s-F	TGACTCAACACGGGAAACC
hRNA18s-R	TCGCTCCACCAACTAAGAAC
hCTGF-F	AGGTAGGAAATGTGGTAGCC
hCTGF-R	AACAAATGCTTCCAGGTGAA
hCYR61-F	CCTCGGCTGGTCAAAGTTAC
hCYR61-R	TTTCTCGTCAACTCCACCTC
hANKRD1-F	GAACTGGTCACTGGAAAGAAG
hANKRD1-R	GGTGGGCTAGAAGTGTCTTC

Supplementary Table 2. List of parameters used in the simulations.

Parameter	Description	Value	References
K_{nuc}	Bulk modulus of nucleus	2.5 kPa	Chosen to match AFM and micropipette measurements of nuclear mechanics. Typical fibroblast nuclei show kPa-scale stiffness[1].
μ_{nuc}	Shear modulus of nucleus	1.2 kPa	
E_{cell}	Elastic modulus of cell at 0.1% strain	2.1 kPa	Typical AFM-measured Young's modulus of fibroblasts at shallow indentations[2].
C_1	Mooney-Rivlin Parameter	0.28 Pa	The Mooney–Rivlin hyperelastic model was used to capture nonlinear cytoskeletal stiffening. C_1 is small relative to C_2, reflecting that compressive stiffening dominates tensile stiffening in the cytoskeleton.
C_2	Mooney-Rivlin Parameter	700 Pa	
ρ	Myosin contractile stress	1.5 kPa	Use of 1–2 kPa is consistent with TFM and modeling estimates for fibroblast contractility[3,4].
η_d	Frictional viscosity constant	90 nN.s/um	Chosen as an effective mesoscale dissipation for many adhesion bonds acting in parallel within fibrillar adhesion. Single slip bond contributes ~1-10 pN per bond, while resisting retrograde flow[4,5] (~0. 01-0.1 um/s). So cluster containing hundreds-thousands of bonds yields a frictional viscosity ~10-100 nN.s/um, consistent with our calibration to the observed hour-scale adhesion disassembly and nuclear recovery dynamics.
S_{focal}	Slope of focal adhesion disassembly curve	-0.04329 int/min	Parameter chosen by doing a linear fit on the experimental data of focal adhesion intensity over time.
$S_{fibrillar}$	Slope of fibrillar adhesion disassembly curve	-0.01467 int/min	Parameter chosen by doing a linear fit on the experimental data of fibrillar adhesion intensity over time.

Supplementary Table 3. Sensitivity analysis of model parameters.

**Parameter**	K_{nuc}	μ_{nuc}	E_{cell}	C_1	C_2	ρ
**Sensitivity**	-0.23874	-0.20809	-0.93311	3.1861e-5	0.092254	1.3802

Supplementary Information References

1. Pogoda, K. *et al.* Unique Role of Vimentin Networks in Compression Stiffening of Cells and Protection of Nuclei from Compressive Stress. *Nano Lett.* **22**, 4725–4732 (2022).
2. Pogoda, K. *et al.* Depth-sensing analysis of cytoskeleton organization based on AFM data. *Eur Biophys J* **41**, 79–87 (2012).
3. Shenoy, V. B., Wang, H. & Wang, X. A chemo-mechanical free-energy-based approach to model durotaxis and extracellular stiffness-dependent contraction and polarization of cells. *Interface Focus* **6**, 20150067 (2016).
4. Balaban, N. Q. *et al.* Force and focal adhesion assembly: a close relationship studied using elastic micropatterned substrates. *Nat Cell Biol* **3**, 466–472 (2001).
5. Alexandrova, A. Y. *et al.* Comparative Dynamics of Retrograde Actin Flow and Focal Adhesions: Formation of Nascent Adhesions Triggers Transition from Fast to Slow Flow. *PLOS ONE* **3**, e3234 (2008).
