# Supplementary material for: Fibrillar adhesion dynamics govern the timescales of nuclear mechano-response via the vimentin cytoskeleton
Source: Nat Mater. 2026 Apr 29;25(7):1252–63. doi: 10.1038/s41563-026-02590-x (PMC13322966; doi:10.1038/s41563-026-02590-x)
Supplement: Supplementary file 2 — Reporting Summary [file 41563_2026_2590_MOESM2_ESM.pdf]

nature portfolio

Corresponding author(s): Amy Beedle
Pere Roca-Cusachs

Last updated by author(s): 31/07/2024

Reporting Summary

Nature Portfolio wishes to improve the reproducibility of the work that we publish. This form provides structure for consistency and transparency in reporting. For further information on Nature Portfolio policies, see our Editorial Policies and the Editorial Policy Checklist.

Statistics

For all statistical analyses, confirm that the following items are present in the figure legend, table legend, main text, or Methods section.

n/a	Confirmed	
☐	☒	The exact sample size (n) for each experimental group/condition, given as a discrete number and unit of measurement
☐	☒	A statement on whether measurements were taken from distinct samples or whether the same sample was measured repeatedly
☐	☒	The statistical test(s) used AND whether they are one- or two-sided<br>*Only common tests should be described solely by name; describe more complex techniques in the Methods section.*
☒	☐	A description of all covariates tested
☐	☒	A description of any assumptions or corrections, such as tests of normality and adjustment for multiple comparisons
☐	☒	A full description of the statistical parameters including central tendency (e.g. means) or other basic estimates (e.g. regression coefficient) AND variation (e.g. standard deviation) or associated estimates of uncertainty (e.g. confidence intervals)
☐	☒	For null hypothesis testing, the test statistic (e.g. F, t, r) with confidence intervals, effect sizes, degrees of freedom and P value noted<br>*Give P values as exact values whenever suitable.*
☒	☐	For Bayesian analysis, information on the choice of priors and Markov chain Monte Carlo settings
☒	☐	For hierarchical and complex designs, identification of the appropriate level for tests and full reporting of outcomes
☐	☒	Estimates of effect sizes (e.g. Cohen's d, Pearson's r), indicating how they were calculated

Our web collection on statistics for biologists contains articles on many of the points above.

Software and code

Policy information about availability of computer code

Data collection	Microscopy images were acquired with; Metamorph (version 7.7.10), Micromanager (version 1.4.22), ZEN (ZEISS, 2.3 SP1 FP3 black version 14.0.24.201), NIS Elements (version 5.42.04). Micropatterns were generated using Leonardo software (version 5.2) via Micromanager (version 2.0.3)
Data analysis	Microscopy images were analysed with ImageJ (version 1.53c). Data statistical tests were performed with Graphpad PRISM (version 9.4.1). Traction force microscopy data were analysed using a custom particle imaging velocimetry software written in Matlab (MathWorks Inc. Version R2019a). Actin anisotropy analysis was performed using the FibrilTool ImageJ plugin. The computational model was implemented in COMSOL Multiphysics. The custom made codes and simulation codes are available on GitHub (https://github.com/vs-vivek/ Nuclear_Mechano_Response).

For manuscripts utilizing custom algorithms or software that are central to the research but not yet described in published literature, software must be made available to editors and reviewers. We strongly encourage code deposition in a community repository (e.g. GitHub). See the Nature Portfolio guidelines for submitting code & software for further information.

Data

Policy information about availability of data

All manuscripts must include a data availability statement. This statement should provide the following information, where applicable:

- Accession codes, unique identifiers, or web links for publicly available datasets
- A description of any restrictions on data availability
- For clinical datasets or third party data, please ensure that the statement adheres to our policy

All values used to generate the graphs in this manuscript are included in the source data file.

Research involving human participants, their data, or biological material

Policy information about studies with human participants or human data. See also policy information about sex, gender (identity/presentation), and sexual orientation and race, ethnicity and racism.

Reporting on sex and gender	No human participants used in this study.
Reporting on race, ethnicity, or other socially relevant groupings	n/a
Population characteristics	n/a
Recruitment	n/a
Ethics oversight	n/a

Note that full information on the approval of the study protocol must also be provided in the manuscript.

Field-specific reporting

Please select the one below that is the best fit for your research. If you are not sure, read the appropriate sections before making your selection.

☒ Life sciences ☐ Behavioural & social sciences ☐ Ecological, evolutionary & environmental sciences

For a reference copy of the document with all sections, see nature.com/documents/nr-reporting-summary-flat.pdf

Life sciences study design

All studies must disclose on these points even when the disclosure is negative.

Sample size	No statistical methods were used to determine the sample size prior to the experiments. Sample sizes for the number of cells analysed for each condition were determined based on previous experience from group members (e.g. Andreu, I., et al. Nat Cell Biol 24, 896–905 (2022). and Kechagia, Z., S. et al. Nat. Mater. 22, 1409–1420 (2023)). The exact sample size for each experimental condition is stated in corresponding figure caption.
Data exclusions	No data were excluded.
Replication	Most data were obtained from a minimum of 3 replicates. In some experiments data were replicated 2 times. The number of replicates for each experiment is explicitly stated in the corresponding figure caption.
Randomization	The cells selected for measurement and analysis were chosen randomly. Furthermore, in experiments with multiple treatments, the cells all originated from the same culture flask and were allocated randomly to each sample.
Blinding	No blinding was carried out because all measurements are quantitative and not biased by the individual. The same individual that performed the experiment conducted the analysis.

Reporting for specific materials, systems and methods

We require information from authors about some types of materials, experimental systems and methods used in many studies. Here, indicate whether each material, system or method listed is relevant to your study. If you are not sure if a list item applies to your research, read the appropriate section before selecting a response.

Materials & experimental systems

n/a	Involved in the study	
☐	☒	Antibodies
☐	☒	Eukaryotic cell lines
☒	☐	Palaeontology and archaeology
☒	☐	Animals and other organisms
☒	☐	Clinical data
☒	☐	Dual use research of concern
☒	☐	Plants

Methods

n/a	Involved in the study	
☒	☐	ChIP-seq
☒	☐	Flow cytometry
☒	☐	MRI-based neuroimaging

Antibodies

Antibodies used

The primary antibodies used for immunofluorescence staining in this study:
YAP (1:300, sc-101199, Santa Cruz) or (1:300, 14074S, Cell Signaling). Integrin α5, clone Snaka51 (1:300, MABT201, Millipore). LaminB (1:300 ab16048, abcam). Paxillin (1:300, ab32084, abcam). Twist (1:100, SC-81417, Santa Cruz). Snail (1:50, Ab224731, abcam). Tensin-1 (1:200, ab233133, abcam). Fibronectin (1:300, F3648, Sigma). Vimentin (1:600, ab92547, abcam). γH2Ax (1:300, 2577, Cell Signaling).

The secondary antibodies used in this study: Alexa Fluor 488 anti-mouse (1:300, A-11029, ThermoFisher), Alexa Fluor 488 anti-rabbit (1:300, A-21206, ThermoFisher), Alexa Fluor 555 anti-rabbit (1:300, A-21429, ThermoFisher), Alexa Fluor 647 anti-rabbit (1:300, A21245, ThermoFisher).

The antibodies used for Western blotting in this study:
Anti-Nesprin3 (ab186746, Abcam, 1:1000). Anti-Vimentin (ab92547, Abcam, 1:2000). Anti-GAPDH (sc-32233, Santa Cruz, 1:1000). Secondary HRP-linked antibody (Jackson ImmunoResearch).

For actin staining: Phalloidin-TRITC (1:1000, P1951-.1mg, Sigma-Alrich) or Phalloidin-iFluor 647 (1:600, ab176759, Abcam).

Validation

All antibodies were used on cells of human origin.
Anti-YAP (mouse monoclonal) was validated for WB, IP, IF, IHC and ELSA in human.
Anti-YAP (rabbit) was validated for WB, IP, IF, and IHC in human.
Anti-Integrin α5, clone Snaka51 was validated for WB, ATC, ICC for human, and validated for IF by independent research articles.
Anti-LaminB was validated for ICC/IF, WB, IHC-P for human.
Anti-Paxillin was validated for ICC/IF, WB, IHC-P for human.
Anti-Twist was validated for WB, IP, IF, FCM for human.
Anti-Snail was validated for HHC-P, ICC/IF for human.
Anti-Tensin-1 was validated by confirming the localisation of adhesions was consistent with Anti-Integrin α5, clone Snaka51 stainings.
Anti-Fibronectin was validated for IF, WB for human.
Anti-Vimentin was validated for ICC/IF, WB, IHC-P for human.
Anti- γH2Ax was validated for WB, ICC/IF for human.

Eukaryotic cell lines

Policy information about cell lines and Sex and Gender in Research

Cell line source(s)

The telomerase immortalised foreskin fibroblast (TIFF) cells were obtained from Johanna Ivaska's lab. The mammary epithelial cells (MCF10A) were purchased from ATCC (Cat # CRL-10317).

Authentication

The cell lines were not authenticated.

Mycoplasma contamination

The cell lines were tested monthly for mycoplasma contamination and were always negative.

Commonly misidentified lines
(See ICLAC register)

Name any commonly misidentified cell lines used in the study and provide a rationale for their use.

Plants

Seed stocks	No plants used in this study.
Novel plant genotypes	*Describe the methods by which all novel plant genotypes were produced. This includes those generated by transgenic approaches, gene editing, chemical/radiation-based mutagenesis and hybridization. For transgenic lines, describe the transformation method, the number of independent lines analyzed and the generation upon which experiments were performed. For gene-edited lines, describe the editor used, the endogenous sequence targeted for editing, the targeting guide RNA sequence (if applicable) and how the editor was applied.*
Authentication	*Describe any authentication procedures for each seed stock used or novel genotype generated. Describe any experiments used to assess the effect of a mutation and, where applicable, how potential secondary effects (e.g. second site T-DNA insertions, mosiacism, off-target gene editing) were examined.*
