# Supplementary material for: Fibrillar adhesion dynamics govern the timescales of nuclear mechano-response via the vimentin cytoskeleton
Source: Nat Mater. 2026 Apr 29;25(7):1252–63. doi: 10.1038/s41563-026-02590-x (PMC13322966; doi:10.1038/s41563-026-02590-x)
Supplement: Supplementary file 8 — Unprocessed western blots for Extended Data Figs. 5g and 6e. [file 41563_2026_2590_MOESM8_ESM.pdf]

Source data for Extended Data Figure 5g.

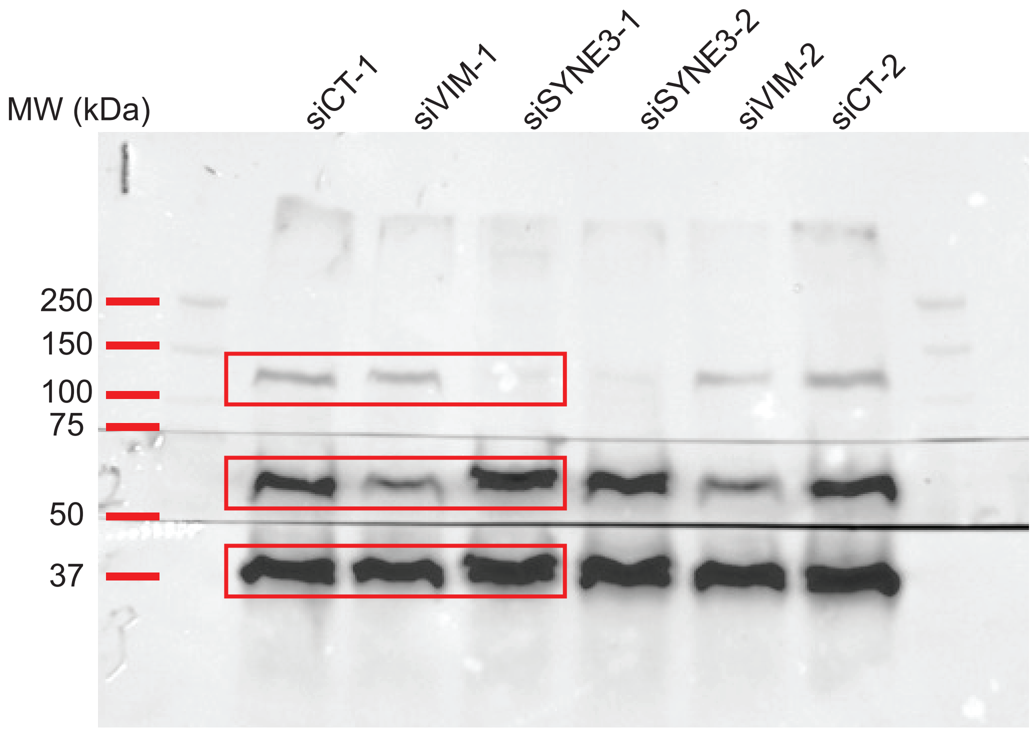

Source data for Extended Data Figure 6e.

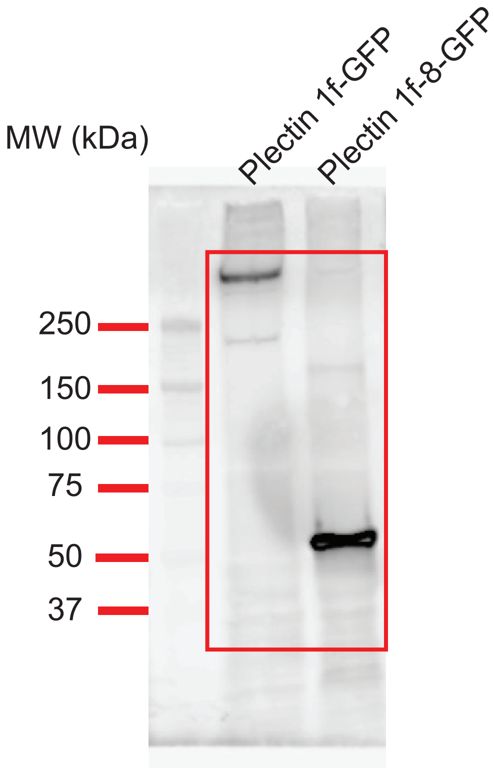

= Cropped region shown in figure

MW = Molecular Weight
